# Vascular endothelial growth factor receptor-1 (VEGFR-1) knock-down is protective against hypoxia, Aβ1-42 oligomer and Aβ1-42 fibril -induced neuronal cell death: implications in AD pathogenesis

**DOI:** 10.3389/fnins.2026.1799391

**Published:** 2026-05-29

**Authors:** Ramcharan Singh Angom, Zonghua Li, Hari Krishnareddy Rachamala, Monica Castanedes-Casey, Tanmay Kulkarni, Trisha Chakravarty, Shamit Dutta, Enfeng Wang, Dennis Dickson, Debabrata Mukhopadhyay, Pritam Das

**Affiliations:** 1 Department of Biochemistry and Molecular Biology, Mayo Clinic Florida, Jacksonville, FL, United States; 2 Department of Neuroscience, Mayo Clinic Florida, Jacksonville, FL, United States

**Keywords:** Alzheimer’s disease, Aβ oligomers/fibrils, hypoxia, neuronal cell death, VEGFR-1

## Abstract

**Introduction:**

Recent transcriptome analysis has demonstrated increased expression of Vascular Endothelial Growth Factor receptor-1 (VEGFR-1/FLT1) and in AD brain. Increased expression of VEGFR1 and its ligand VEGFB were associated with a more rapid rate of cognitive decline, providing evidence of a potential link between increased VEGFR-1 expression in AD pathogenesis. In this study, we explored the potential role of VEGFR-1 expression in neurons on AD pathology.

**Methods:**

To confirm VEGFR1 expression in AD brains, we first performed immunostaining in AD brain sections (AD - Braak stage V-VI, and normal controls - Braak 0-II). And to determine a potential detrimental role of neuronal VEGFR1 expression on AD associated pathologies, we exposed SH-SY5Y human neuroblastoma cells and mouse primary neurons to either hypoxia conditions (1%O_2_) or 5 μ Aβ1-42 oligomers or fibrils for 24, 28 and 72hrs.

**Results:**

In this study, we found preferential staining of VEGFR-1 in the neuropil and neuronal cell bodies both in AD and Control hippocampus and increased VEGFR-1 immunoreactivity in dystrophic neuritic processes in the vicinity of Thio-S positive amyloid plaques in AD brains. And treatment of SH-SY5Y human neuroblastoma cell line and mouse primary neurons, with either hypoxia conditions or Aβ1-42 oligomers, resulted in increased VEGFR-1 expression and cleaved caspase 3 activation, leading to neuronal toxicities/cell death. Similarly, treatment with Aβ1-42 fibrils also increased VEGFR-1 and cleaved caspase 3 protein levels in the SH-SY5Y cells whereas treatment with Aβ1-42 monomers had no effect on VEGFR-1 expression. In addition, we show that over-expression of VEGFR-1 intracellular domains in SH-SY5Y cells directly induced neuronal toxicities and importantly, siRNA-mediated knockdown of VEGFR-1 in neurons prevented the hypoxia, Aβ1-42 oligomer and Aβ1-42 fibril-induced toxicities and cell death phenotypes. Treatment with either hypoxia or Aβ1-42 oligomers also reduced expression of cell survival genes including VEGFR-2 and Hippo pathway YAP1 and siRNA-mediated VEGFR-1 knockdown in the neurons normalized expression of both VEGFR-2 and YAP1. Using differential gene expression analysis, we demonstrated upregulation of several inflammatory/interferon-stimulated genes (ISGs) as well as increased expression of genes involved in activation of oxidative stress and cell death pathways in response to Aβ1-42 oligomers treatment in mouse primary neurons. And siRNA-mediated VEGFR-1 knockdown in the mouse primary neurons, reduced gene expression of both the ISGs and oxidative stress/cell death pathways in response to Aβ1-42 oligomer treatment.

**Discussion:**

In summary, these results show that siRNA-mediated knockdown of VEGFR-1 in neurons significantly prevented hypoxia, Aβ1-42 oligomer and Aβ1-42 fibril-induced cellular toxicities and cell death phenotypes, indicating a potential detrimental role of aberrant VEGFR-1 expression and signaling in response to AD associated pathologies.

## 1 Introduction

The vascular endothelial growth factor (VEGF) and their receptors (VEGFRs) are well known for their important physiological roles in angiogenesis and endothelial cell survival, migration, and proliferation (Ferrara et al., 2003; Cao, 2009; Ruiz de Almodovar et al., 2009; Carmeliet and Ruiz de Almodovar, 2013; Lange et al., 2016; Simons et al., 2016; Perez-Gutierrez and Ferrara, 2023). The VEGF family members include multiple growth factors including VEGFA, VEGFB, VEGFC, VEGFD, and Placental Growth Factor (PlGF) that signal through specific receptor tyrosine kinases including VEGFR-1 (*FLT1*), VEGFR-2 (*KDR*), and co-receptors Neuropilin 1 (NRP1) and NRP2 (Ferrara et al., 2003; Cao, 2009; Ruiz de Almodovar et al., 2009; Carmeliet and Ruiz de Almodovar, 2013; Lange et al., 2016; Simons et al., 2016; Perez-Gutierrez and Ferrara, 2023). Although the role of VEGFR-2 in endothelial cell survival and proangiogenic signals has been extensively studied, the precise role of VEGFR-1 signaling is poorly understood (Fong et al., 1995; Zeng et al., 2001; Carmeliet, 2003; Cao, 2009). Increases in VEGFR-1, specifically its alternatively spliced soluble/decoy version (sVEGFR1), (which has ∼10-fold higher affinity to bind VEGFA compared to VEGFR-2 and can efficiently block VEGFA-VEGFR2 signaling (Risau, 1997) was recently shown to have detrimental effects on aging-related phenotypes in mice (Grunewald et al., 2021). Genetic deletion of VEGFR-1 knock out in mice showed disorganization of blood vessels and died *in utero* between E8.5 and E9.0, suggesting that VEGFR1 may be a negative regulator of VEGFR2 as well as the angiogenesis process in endothelial cells (Fong et al., 1995). Although studies in AD mouse models have also shown that treatment with exogenous VEGFA or Epidermal growth factor (EGF) can ameliorate memory impairment and improve cerebrovascular functions (Cao et al., 2004; Licht et al., 2011; Wang et al., 2011; Religa et al., 2013; Garcia et al., 2014; Koster et al., 2016; Thomas et al., 2016; Martin et al., 2021), and have beneficial microglial phagocytic responses to Aβ peptides (de Gea et al., 2023), the role of VEGF/VEGFR signaling in AD brain remain contradictory as evidence of both up- and down-regulation of VEGF gene and protein expression in the brain parenchyma, cerebrospinal fluid (CSF) and in peripheral blood has been reported in AD patients (Tarkowski et al., 2002; Chiappelli et al., 2006; Corsi et al., 2011; Craig-Schapiro et al., 2011; Hohman et al., 2015; Cho et al., 2017; Ceci et al., 2024; Yang et al., 2024). Thus, a more precise understanding of the interactions between VEGF ligands and their receptors in AD brain is warranted.

In this regard, recent transcriptome analysis data using single cell/nuclei analysis has shed more insight into the potential role of VEGF family members including both the VEGF receptors and their ligands on AD pathogenesis. A previous report using single cell RNA-seq transcriptome analysis revealed that brain endothelial cells (ECs) in AD brain showed increased expression of angiogenic growth factors and their receptors including VEGFR-1/FLT1 and other immune markers compared to controls, suggesting an abnormal angiogenic state in the AD brain ECs (Lau et al., 2020). Another report (using bulk RNA sequencing of AD prefrontal cortex tissue) convincingly demonstrated that increased expression of VEGFR-1 and its ligands, including placental growth factor (PIGF) and VEGFB, associated with a more rapid rate of cognitive decline in AD samples (Mahoney et al., 2019). In recent follow up papers by the same group, using single cell nuclei analysis as well as tandem mass tag and selected reaction monitoring mass spectrometry proteomic measures from the post-mortem brains, they confirmed increased expression of VEGFR-1 and VEGFB in various cell types including neurons, microglia, oligodendrocytes, and endothelial cells (ECs) and demonstrated higher expression of VEGFR-1 associated with worse disease outcomes (Seto et al., 2023; Wu et al., 2025) Similarly, using a novel pathway-based statistical approach, another study demonstrated vascular endothelial growth factor receptor binding (VEGF-RB) expression associated with amyloid-β and tau pathology and worsening cognition in AD (Mahzarnia et al., 2024). This report also confirmed higher expression of VEGFR-1/FLT1 and its ligands including VEGFB and lower expression of VEGFR-2 (KDR), VEGFR-3 (FLT4), NRP1, and NRP2, in AD samples compared to controls, mirroring findings from the above-mentioned reports. Lastly, another recent study also demonstrated increased immunoreactivity of VEGFR-1/FLT1 and other novel proteins in neuritic processes near amyloid plaques in aged APP CRND8 mice (Levites et al., 2024).

These recent data have highlighted a potential detrimental role of increased VEGFR-1 expression and signaling events in AD pathogenesis. While the role of VEGFA- VEGFR-2 signaling in endothelial cell proliferation, survival and proangiogenic signals (Fong et al., 1995; Zeng et al., 2001; Ferrara et al., 2003; Cao, 2009; Ruiz de Almodovar et al., 2009; Koch and Claesson-Welsh, 2012; Carmeliet and Ruiz de Almodovar, 2013; Lange et al., 2016; Perez-Gutierrez and Ferrara, 2023), and neuroprotection functions in AD mouse models (Cao et al., 2004; Licht et al., 2011; Wang et al., 2011; Religa et al., 2013; Koster et al., 2016; Thomas et al., 2016) has been extensively studied, the potential role of aberrant VEGFR-1 expression and signaling in AD pathogenesis is not well known. In the current study, we examined the role of VEGFR-1 expression on AD pathology, and our data shows preferential neuronal staining of VEGFR-1 in AD brain hippocampus with increased immunoreactivity in dystrophic neuritic processes in the vicinity of amyloid plaques. Secondly, using cultured neuronal cells, treatment with either hypoxia conditions, Aβ1-42 oligomers or Aβ1-42 fibrils, led to increased VEGFR-1 expression and activation of neuronal toxicities/cell death. Importantly, VEGFR-1 knock down in the neurons prevented the hypoxia, Aβ1-42 oligomer and fibril induced neuronal toxicities/cell death. These data demonstrate that increased VEGFR-1 expression and signaling events may contribute to detrimental effects in neurons and suggest that modulation of VEGFR-1 expression and signaling in the neurons may have beneficial effects on AD associated pathologies.

## 2 Materials and methods

### 2.1 Brain samples

Post-mortem brain Tissue was obtained from the Florida Brain Bank of Neurodegenerative Disorders at Mayo Clinic that had previously undergone neuropathological assessments, including Braak staging and cerebrovascular pathology scoring. For our immuno-histological studies, we selected 10 neuropathologically confirmed sporadic AD cases (Braak stage V-VI, Thal phase ≥ 3, Ave. Age = 79 ± 5.7) and 10 neurologically normal elderly subjects without significant AD pathology (Braak 0-II, Thal ≤ 1, Ave. Age = 73 ± 1.4).

### 2.2 Immunohistochemistry

Five microgram paraffin-embedded sections of AD samples and control brain tissue (from hippocampus) and 15-month-old Tg2576 mouse brains were deparaffinized and rehydrated with deionized water (dH_2_O) as follows: Three changes of xylene 5 min each, two changes of 100% alcohol 3 min each, and one change of 95% alcohol for 3 min, then rinse well in dH20. Then antigen retrieval was performed by streaming the slides for 30 min with either of the following solutions: a. Deionized water; b. Citrate buffer pH6; or c. Tris-EDTA pH9. After steaming, the slides were cooled under running deionized water. And all subsequent immunostaining steps were performed using an Autostainer (Thermo Scientific Lab Vision Autostainer 480S, Carlsbad, CA, United States) at room temperature as follows; Peroxidase block 5 min, 5% Normal goat serum -20 min, Primary antibody-45 min, Secondary antibody -30 min (Anti-Mouse or Anti-Rabbit Labeled Polymer-HRP, DAB + Chromogen -5 min, Deionized water rinse -3 min and then counterstained for 2–10 s in Shandon Gill 1 Hematoxylin. The following Primary antibodies were used: 1. Anti-VEGFR-1 (1:50, H2O steam, Y-103, Rabbit monoclonal, Abcam, Waltham, MA, United States); 2. Anti-VEGFR-1 [1:200, Citrate buffer pH6, Sy09-09, Rabbit Monoclonal, Novusbio, Centennial CO, United States); 3. Anti-VEGFR-1 (1:200, Tris-EDTA pH9, CL0345, mouse monoclonal, Novusbio, Centennial CO, United States)]; 4. Anti-VEGFR-1 [1:200, Tris-EDTA pH9, ab2350, Rabbit polyclonal, Abcam, Waltham, MA, United States; 5. Anti-Ubiquitin (1:50,000, H2O steam, Ubi-1, mouse Monoclonal, EMD Millipore, Burlington, MA, United States)]. And the following reagents were used: 1. Dako Target Retrieval Solution (10X Citrate pH6), DAKO Code S1699; 2. Dako Target Retrieval Solution (10X Tris-EDTA pH9), DAKO Code S2367; 3. Antibody Diluent with background reducing components, DAKO Code S3022; 4. EnVision + System-HRP Labeled Polymer Anti- mouse, DAKO Code K4001; 5. EnVision + System-HRP Labeled Polymer Anti-Rabbit, DAKO Code K4003. All Dako reagents were purchased from Agilent Technologies Santa Clara, CA, United States).

### 2.3 Single-nuclei RNA sequencing

The single-nuclei RNA sequencing data from Mathys et al. (2019) were generated using frozen postmortem prefrontal cortex samples from 24 control cases and 24 Alzheimer’s disease (AD) cases. The associated expression matrix and metadata were available for download at under Synapse ID syn21788402. Ambient RNA contamination was estimated using the decontX function from the celda package (v.1.12.0). Doublets and multiplets were computationally identified using DoubletFinder (v.2.0.3) and were excluded from the analysis. Additionally, cells exhibiting a mitochondrial gene percentage exceeding 5%, with fewer than 200 total features, or with features expressed in fewer than 5 cells were excluded from downstream analyses. Cell clustering was performed using Uniform Manifold Approximation and Projection (UMAP) with the first 30 principal components derived from the top 3,000 most variable genes. Clusters were annotated based on marker genes identified using two-part hurdle models implemented in the FindAllMarkers function of the Seurat R package (v.3.1.0). Differential gene expression analysis between AD and normal control (NC) groups was conducted using two-part hurdle models, adjusted for age at death, sex, and gene expression rate, with sample ID as a random effect. The single-nuclei data were normalized using the LogNormalize function in Seurat (v4.3.0.1) for visualization.

#### 2.3.1 Aβ1-42 oligomers preparation

For these studies, we have utilized a well characterized low molecular weight Aβ1-42 oligomer species (2–4 mer originally developed by the M.J. Ladu group (Dahlgren et al., 2002). We generated Aβ1-42 oligomer species using similar recently described protocols (Cakir et al., 2019) as follows: Briefly, Aβ1-42 monomers were thawed at room temperature and transferred to a glass vial followed by flash freezing on dry ice. The vial was then kept in a lyophilizer overnight, and the next day the lyophilized monomer was mixed with 250 μL HIFP to bring the final concentration to 225 μM. The vial was again lyophilized overnight, and a thin film was observed which was dissolved in DMSO, sonicated for 10 min and then the mixture was dissolved in 200 μL phenol red free medium and incubated at 4°C overnight to form oligomers, and immediately used for experimentation. We confirmed using Atomic Force Microscopy (AFM) that this Aβ1-42 oligomeric species appears as small globular structures (see Supplementary Figures 1A–C). To obtain Aβ1-42 fibril samples, Aβ1-42 monomer (1 mg/mL) was incubated in PBS buffer at 37°C for 24 h under quiescent conditions. We then confirmed using AFM that the Aβ1-42 fibrils appear as fibrillar structures (see Supplementary Figure 1D).

#### 2.3.2 Neuronal cell culture

The following 2 neuronal cells lines were used: 1. SH-SY5Y human neuroblastoma cell line: Undifferentiated SH-SY5Y cells were seeded in growth medium (DMEM medium (10% FBS, 2% GlutaMAX and 1% pen/strep) at 37°C, 5% CO_2_ for 24–48 h and then cells were treated with either Aβ1-42 oligomers (5 μM) or Aβ1-42 fibrils (5 and 10 μM) or hypoxia conditions (1% O_2_ or CoCL_2_, 200 μM) for an additional 24, 48, and 72 h and then cell lysates were used western blotting or qPCR analysis; 2. Mouse primary neurons (Mouse CD1 Brain Cortex Neurons, Cat # M-CX-400, Lonza, Morristown, NJ, United States), which were cultured as per manufactures protocols. Briefly, the mouse primary neurons were thawed and plated on Poly lysine-D treated 6-well culture plates, and then cultured using PNBM™ Basal Medium (Lonza, CC-3256) and PNGM™ Singlequots™ Growth Supplements (Lonza, CC-4462) for 2 weeks, when abundant neurite networks were visible. The cells were then treated with Aβ1-42 oligomers (5 μM) for 24, 48, and 72 h and then cell lysates were used western blotting or qPCR analysis.

#### 2.3.3 MTT assays

Neuronal viability/cell death in SH-SY5Y cells and mouse primary neurons was measured using MTT assays (Promega, Madison, WI, United States) as described previously (Kulkarni et al., 2019; Singh Angom et al., 2019). Briefly, 5 × 10^3^ cells were seeded in the 96-well plates in growth medium and then exposed to either hypoxia conditions or Aβ1-42 oligomers/Fibrils for 24, 48, and 72 h, and neuronal viability was measured using MTT assay.

#### 2.3.4 Apoptosis assays

SH-SY5Y cells were seeded in growth medium (DMEM medium (10% FBS, 2% GlutaMAX and 1% pen/strep) at 37°C, 5% CO_2_. For gene silencing experiments, cells were transfected with VEGFR1-specific siRNA or control siRNA using Lipofect, RNAiMAX (Invitrogen). 24 h after transfection, cells were then treated with Aβ1-42 oligomers (5 μM) for an additional 72 h and apoptotic cells were detected using 1. *Apopxin Green Indicator*, which selectively binds phosphatidylserine on apoptotic cells. Following treatments, cells were incubated with Apopxin reagent according to the manufacturer’s instructions (Abcam, ab176749), washed, and imaged using a confocal laser scanning microscope (Zess LSM880). Live cells were also counterstained with a Calcein violet for quantification. Images were acquired under identical settings using 10x objective for all groups. Fluorescence intensity was quantified using image J analysis software (5 images/group), and apoptotic signals were expressed as fold change relative to control; 2. *Annexin V*- Apoptosis was quantified using the Alexa Fluor 488 Annexin V/Dead Cell Apoptosis Kit (Invitrogen). Following treatment, cells were harvested, washed with cold PBS, and stained with Alexa Fluor 488 Annexin V and propidium iodide (PI) according to the manufacturer’s protocol. Briefly, cells were resuspended in Annexin-binding buffer and incubated with Annexin V and PI for 15 min at room temperature in the dark. Samples (*n* = 3 samples/group) were analyzed using an Attune Nx Flow Cytometer (Thermo Fisher Scientific). Data were processed to distinguish viable (Annexin V^–^/PI^–^), early apoptotic (Annexin V^+^/PI^–^), late apoptotic (Annexin V^+^/PI^+^), and necrotic (Annexin V^–^/PI^+^) cell populations and the percentage of total apoptotic cells (early + late apoptosis) was calculated for each condition.

### 2.4 Western blotting

After incubation with hypoxia or Aβ1-42 oligomers/fibrils, the protein lysates were prepared using NP40 lysis buffer (Cat #J60766, Thermo Scientific) with protease and phosphatase inhibitor (Cat #B14001, B15001, Selleckchem). The following antibodies were used: VEGFR-1 (rabbit pAb, 2893S) and VEGFR-2 (rabbit polyclonal, 2479S) from Cell Signaling Technology, Danvers, Massachusetts, United States); cleaved caspase-3 (#9661) from Cell signaling technology (Danvers, Massachusetts, United States); YAP1 (#4912) from Cell signaling technology (Danvers, Massachusetts, United States);The anti-β actin antibody was obtained from MilliporeSigma (Burlington, MA, United States). Following primary and secondary antibody incubation, the membranes were developed using ECL detection system (Bio-Rad, Hercules, CA, United States).

### 2.5 RT-PCR

For mRNA expression analysis, total RNA was extracted using RNeasy Kit (Qiagen, Germantown, MD, United States). 1 μg of total RNA was reverse transcribed using an iScript cDNA Synthesis Kit (Bio-Rad) as described in the manufacturer’s protocol. The PCR was performed using 0.1 μg of cDNA in a 10 μL of PCR mix containing 500 nM of each primer power sybr master mix (Life Technologies, Carlsbad, CA, United States), and the reaction was performed using 7,500 PCR system (Applied Biosystems) (40 cycles of amplification at 95°C for 15 s and 57°C for 1 min). The PCR primer sequences were as follows:

VEGFR-1 “Forward-TCTCACACATCGACAAACCAATACA;
Reverse- GGTAGCAGTACAATTGAGGACAAGA”
VEGFR-2 “Forward-GCAGGGGACAGAGGGACTTG; Reverse- GAGGCCATCGCTGCACTC”
YAP1 “Forward-TAGCCCTGCGTAGCCAGTTA; Reverse-TCATGCTTAGTCCACTGTCTGT”

### 2.6 VEGFR-1 Si RNA transfection

The VEGFR-1 siRNA sequences were characterized and validated as previously described (Angom et al., 2022). For VEGFR-1 knockdown, the neurons were seeded in 6-well plates and transfected with 50 nM VEGFR1-targeting siRNA or non-targeting control siRNA (24 h prior to hypoxia or Aβ1-42 oligomer or fibril) treatment using Lipofectamine Transfection Reagent, (RNAiMAX, Invitrogen) as per manufacturers protocol.

### 2.7 VEGFR-1 “EGLT” transfection

The VEGFR-1 “EGLT” chimeric construct consisting of the extracellular domain of EGFR and the transmembrane and intracellular domains of VEGFR-1 was generated and packaged in Retrovirus as previously described (Zeng et al., 2001; Singh Angom et al., 2019). The “EGLT” containing Retrovirus was used for transfection as follows: SH-SY5Y cells were seeded at a density of 3 × 10^6^ cells in a 100-mm plate for 24 h and 5 mL of retrovirus solution and 5 mL of fresh medium were added to the cells with 10 μg/mL polybrene. The media was changed after 16 hr, and cells were then analyzed 24, 48, and 72 h after transfection for neuronal viability using MTT assays.

### 2.8 Differential gene expression analysis

For differential gene expression analysis, the mouse primary neurons (Mouse CD1 Brain Cortex Neurons, Cat # M-CX-400, Lonza, Morristown, NJ, United States) were treated with Aβ1-42 oligomer (5 μM) for 24 h; For knockdown experiments, the neurons were transfected with 50 nM VEGFR-targeting siRNA or non-targeting Control siRNA using Lipofectamine (RNAiMAX, Invitrogen) 24 h prior to Aβ1-42 oligomer treatment as described above and then the cells were flash frozen in liquid nitrogen and shipped for RNA extraction and sequencing at MedGenome Inc. (Foster City, CA, United States). The RNA quantity was measured using a nanodrop, and the RNA integrity was verified using the Agilent 2100 Bioanalyzer, and only samples with RNA Integrity Number (RIN) > 8 were processed further. Library preparation was performed using the Illumina TruSeq Stranded mRNA Library Prep Kit and paired-end sequencing (150 bp reads) were conducted at MedGenome Inc., Foster City, CA, United States. Raw sequencing data were quality-checked using FastQC, and adapters and low-quality reads were removed using Trimmomatic. High-quality reads were aligned to the mouse reference genome (GRCm38/mm10) using the STAR aligner. Gene-level quantification was performed using featureCounts, and differential gene expression analysis was carried out using the DESeq2 package in R. Genes with an adjusted *p*-value < 0.05 and an absolute log2 fold change ≥ 1 were considered significantly differentially expressed. Gene ontology (GO) and KEGG pathway enrichment analyses were performed using clusterProfiler and DAVID to identify biological processes and pathways differentially regulated across the treatment groups. The following groups were used for analysis—Control, Aβ1-42 oligomers only and Aβ1-42 oligomers + VEGFR1 siRNA).

### 2.9 Statistical analysis

Statistical analyses were performed with the Graphpad Prism 7 software (SPSS Inc.). Comparisons between 2 groups were done by Student’s *t-*test and one-way ANOVA was used for multi-group comparisons. For the gene expression analysis, differential expression analysis was performed using the DESeq2 package (v1.38.3)^1^ (R Bioconductor package in R). Raw gene count data were normalized using the median-of-ratios method to control for sequencing depth and compositional differences across samples. Genes with *p*-value (FDR) < 0.05 and an absolute log2 fold change ≥ 1 were considered significantly differentially expressed. For more accurate estimation of log_2_ fold changes, shrinkage was applied (Love et al., 2014), which improves interpretability and controls variability in low-count genes. Principal component analysis (PCA) and hierarchical clustering were used to evaluate sample separation and global expression trends across treatment groups.

## 3 Results

### 3.1 VEGFR-1 immunoreactivity is predominantly detected in the neuropil and in neuronal cell bodies with increased immunoreactivity near amyloid plaques

**FIGURE 1:**
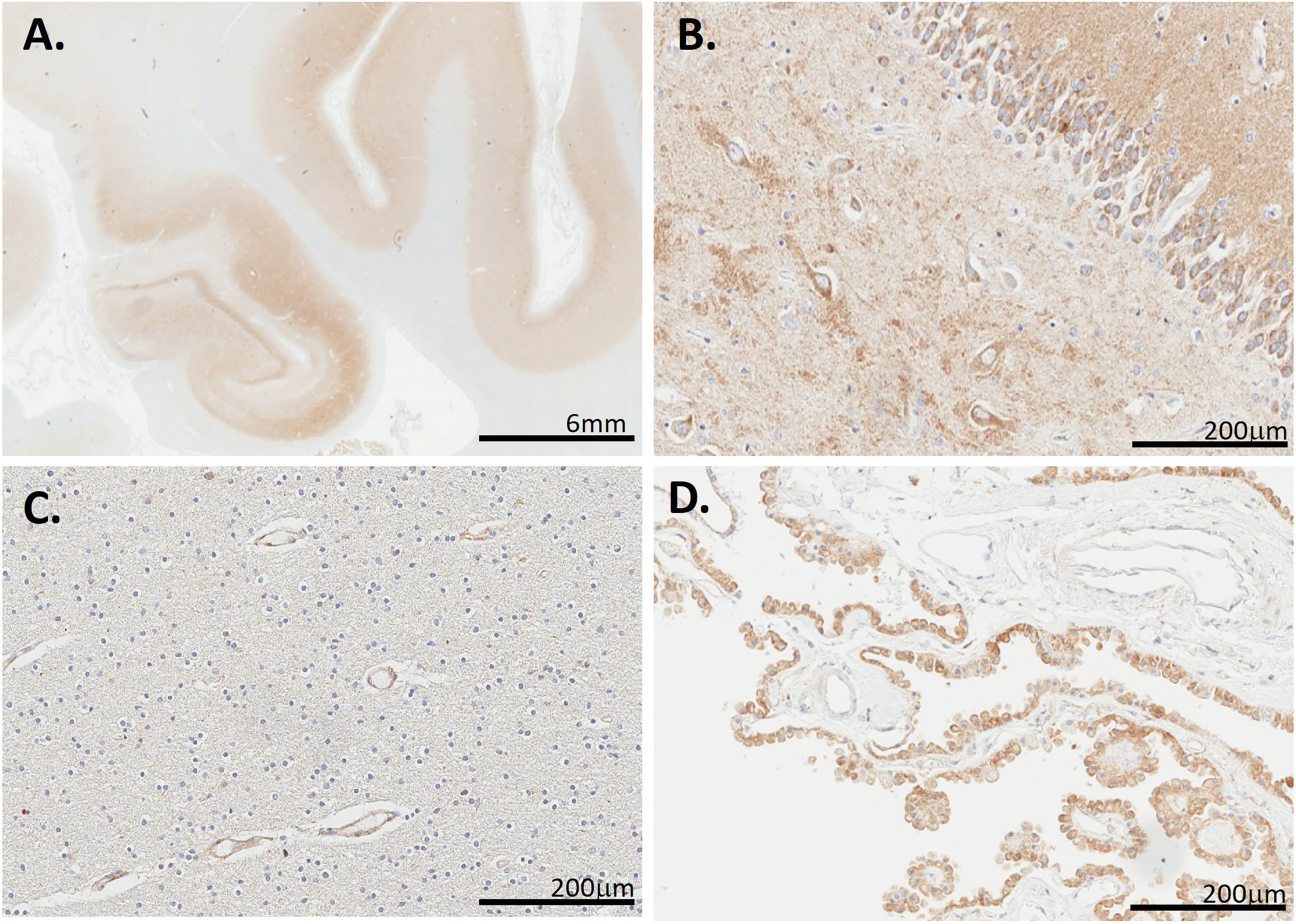
Cellular staining of VEGFR-1 in AD brain. **(A)** Representative lower magnification image of VEGFR-1 immunostaining (using Anti-VEGFR-1 antibody #Y-103 N’ terminal epitope, Abcam) in AD brain hippocampus and **(B)** higher magnification image from **(A)** showing VEGFR-1 staining in the neuropil and neuronal cell bodies in AD hippocampus. **(C)** Representative image showing VEGFR-1 staining in ECs in white matter areas of AD brain. **(D)** Representative image showing VEGFR-1 staining of Ependymal cells lining the ventricles in AD brain.

**FIGURE 2:**
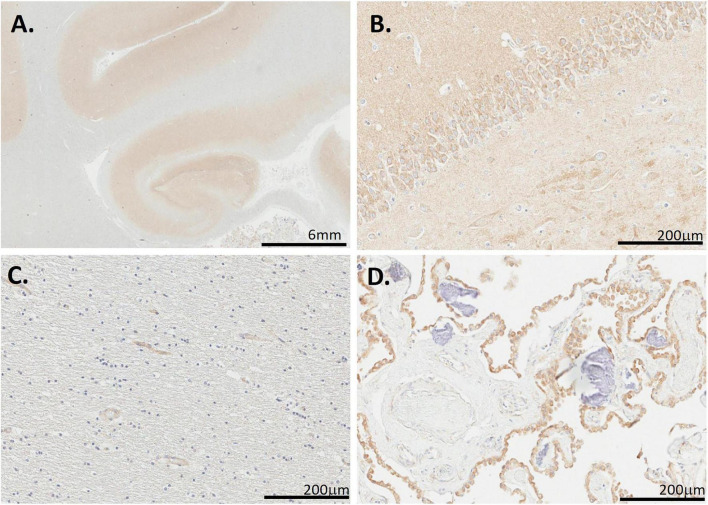
Cellular staining of VEGFR-1 in Normal control brain. **(A)** Representative lower magnification image of VEGFR-1 immunostaining (using Anti-VEGFR-1 antibody, Y-103 N’ terminal epitope, Abcam) in Normal control hippocampus and **(B)** higher magnification image from **(A)** showing VEGFR-1 staining in the neuropil and neuronal cell bodies in the Normal hippocampus. **(C)** Representative image showing VEGFR-1 staining in ECs in white matter areas of Normal brain. **(D)** Representative image showing VEGFR-1 staining of Ependymal cells lining the ventricles in Normal brain.

To identify the cellular sources and expression patterns of VEGFR-1 in AD brains, we performed anti-VEGFR-1 immuno-staining using paraffin-embedded fixed AD brain sections (AD *n* = 10 (Braak stage V-VI, and normal controls *n* = 10 Braak 0-II) using multiple anti-VEGFR-1 antibodies. Using the same antibody described in a previous study (Harris et al., 2018) (anti-VEGFR1, #Y-103 N’ terminal epitope, Abcam), our analysis readily detected VEGFR-1 staining in the neuropil and in neuronal cell bodies in the hippocampus both in AD samples (Figures 1A,B) and age-matched Control samples (Figures 2A,B). Although robust immunoreactivity was observed in AD brain neuropil compared to Control samples, quantification of the immunoreactivity was not significant *(data not shown*). In addition, this antibody showed weak immunostaining of ECs, (more readily visible in white matter areas, away from the neuropil) both in AD samples (Figure 1C) and Controls (Figure 2C) and also robustly stained Ependymal cells lining the ventricle both in AD brain samples (Figure 1D) and Controls (Figure 2D).

**FIGURE 3:**
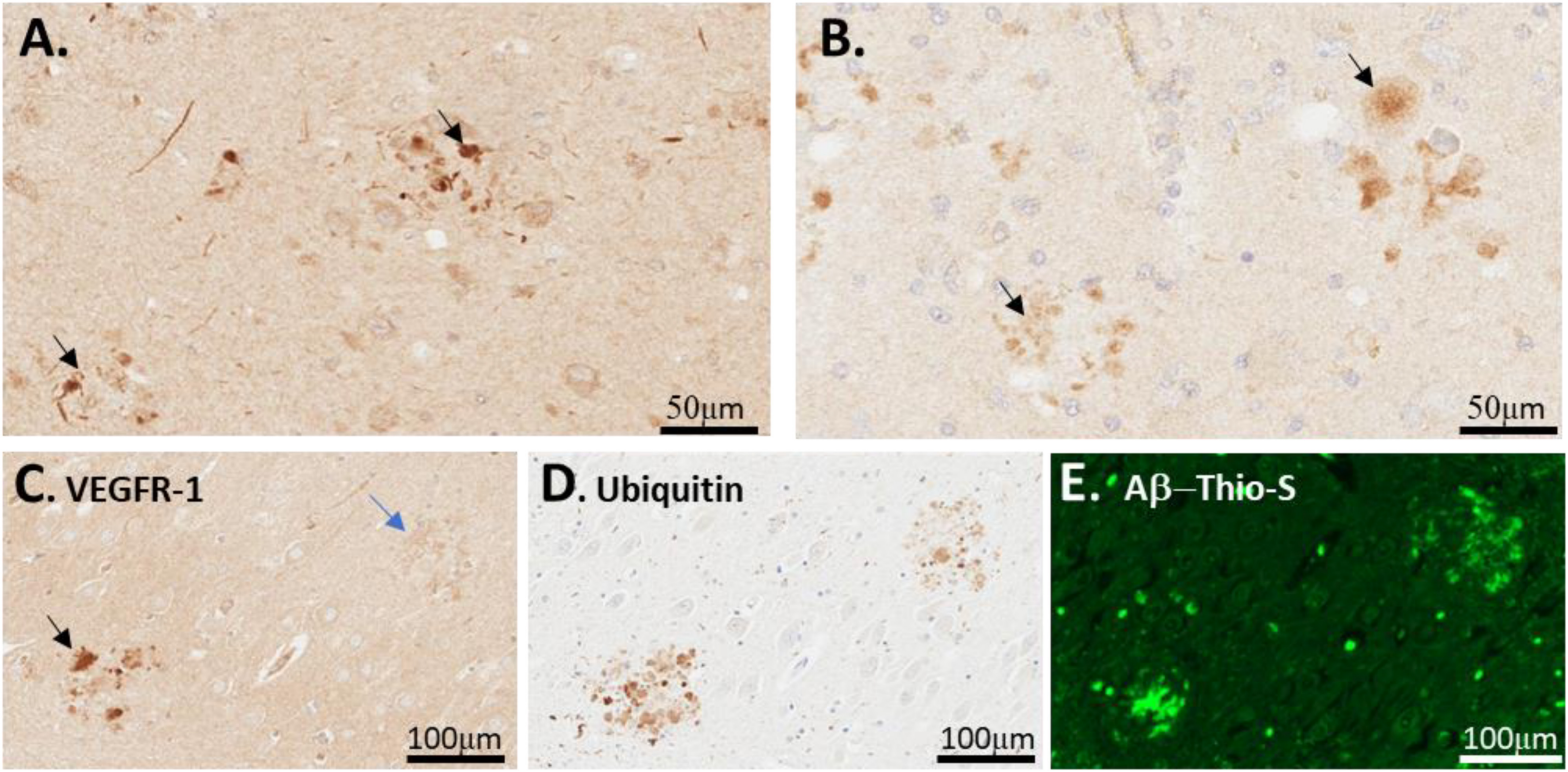
Increased VEGFR-1 staining in neuritic processes/axonal swellings near amyloid plaques in AD brain. **(A)** Representative image of VEGFR-1 immunoreactivity in dystrophic neuritic processes near amyloid plaques (black arrows, using Anti-VEGFR-1 antibody, C’-Ter epitopes, Ab2350, Abcam) in AD brain and **(B)** representative image of VEGFR-1 immunoreactivity in dystrophic neuritic processes near amyloid plaques (black arrows, using anti-VEGFR-1 antibody C’-Ter epitopes, CL0345, Novus Bio) in AD brain. Using serial sections, we show; **(C).** Increased immunoreactivity of VEGFR-1 (black arrow, using anti-VEGFR-1 antibody C’-Ter epitopes, CL0345, Novus Bio). **(D)** In ubiquitin positive dystrophic neurites/axonal swellings specifically near. **(E)** Thio-s positive “cored” amyloid plaques (lower left-(black arrow in **C**) but not near “diffuse” amyloid plaque (upper right- (blue arrow in **C**).

**FIGURE 4:**
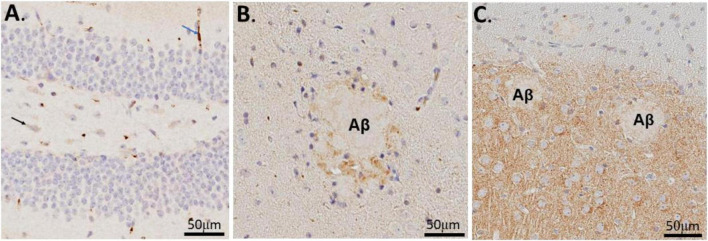
Cellular staining of VEGFR-1 in 15-month-old Tg2576 mouse brain. **(A)** Representative image of VEGFR-1 staining (using anti-VEGFR-1 antibody, C’ Ter epitopes, CL0345, Novus Bio) in neurons (black arrow) and EC/microvessel (blue arrow) in hippocampus and **(B)** VEGFR-1 staining near “cored” amyloid plaques (“Aβ,” using anti-VEGFR-1 antibody CL0345, C’ Ter epitopes, Novus Bio). **(C)** VEGFR-1 staining (using anti-VEGFR-1 antibody SY09-09, N’-Ter epitopes, Invitrogen) in neurons and the neuropil near amyloid plaques (“Aβ”).

Interestingly, using two different anti-VEGFR-1 antibodies with *C-terminal epitopes* (Mab CL0345-Novus Bio and Ab2350-Abcam), we did not observe VEGFR-1 immunostaining in the neuropil, but rather these anti-VEGFR-1 antibodies showed increased immunoreactivity specifically in the vicinity of amyloid plaques (Figures 3A,B). Using serial sections, we confirmed this increased VEGFR-1 immunoreactivity (using anti-VEGFR-1 antibody Mab CL0345) in the vicinity of ubiquitin positive neuritic processes/axonal swellings, specifically near “cored” Thio-S positive amyloid plaques but not near “diffuse” amyloid plaques (Figures 3C–E). Using the same anti-VEGFR-1 C-terminal antibody (Mab CL0345-Novus Bio) we also confirmed VEGFR-1 immunostaining in neuronal cell bodies and strong immunoreactivity in brain ECs (Figure 4A) and increased VEGFR-1 immunoreactivity in neuritic processes in the vicinity of “cored” amyloid plaques in aged 15-month-old Tg2576 mouse hippocampus brain sections (Figure 4B). Similar strong immunoreactivity in the neuropil and in neuronal cell bodies was also observed in the 15-month-old Tg2576 hippocampus brain sections (Figure 4C) using an anti-VEGFR-1 antibody with the N-Terminal epitopes (SY09-09, Invitrogen). Collectively these immunohistology results demonstrate VEGFR-1 expression predominantly in the neuropil and neurons both in the AD brain and Controls, with increased immunoreactivity in neuritic processes near Thio-S positive “cored” amyloid plaques in AD brain samples.

**FIGURE 5:**
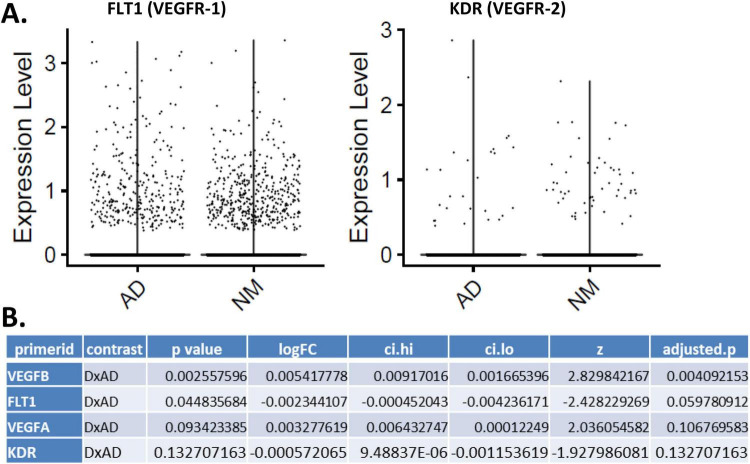
Single cell RNA-seq analysis of FLT/(VEGFR1), KDR(VEGFR2), VEGFA and VEGFB in neurons from AD brain (AD) and Normal controls (NM). **(A)** Rplot of FLT1/VEGFR-1 and KDR/VEGFR-2 expression. **(B)** Statistical analysis.

We next queried a previously published RNA-seq dataset (Mathys et al., 2019) to quantify and further analyze VEGFR-1/FLT1 expression in AD neurons (we generated expression data from both excitatory and inhibitory neurons for this analysis). We confirmed neuronal expression of VEGFR-1/FLT1 both in AD and Normal control brains (Figures 5A,B), (combined analysis is shown and expression levels in Normal controls were used as the reference level) and although there was a trend in increased VEGFR-1 expression in AD neurons compared to Normal, this effect was not significant. We also show minimal/low expression of VEGFR-2/KDR both in AD and Normal control neurons (Figures 5A,B), similar to the results from cell culture studies, which show low VEGFR-2 expression/protein levels in the cultured neurons. And although no significant changes in neuronal expression levels of VEGFA were observed, significantly increased expression levels of VEGFB were seen in AD neurons compared to Normal controls, mirroring findings regarding increased VEGFB from the recently published RNA seq data in AD brains (Mahoney et al., 2019; Lau et al., 2020; Seto et al., 2023; Mahzarnia et al., 2024).

### 3.2 VEGFR-1 Knock-down is protective against hypoxia, Aβ1-42 oligomer and Aβ1-42 fibril induced neuronal cell death

**FIGURE 6:**
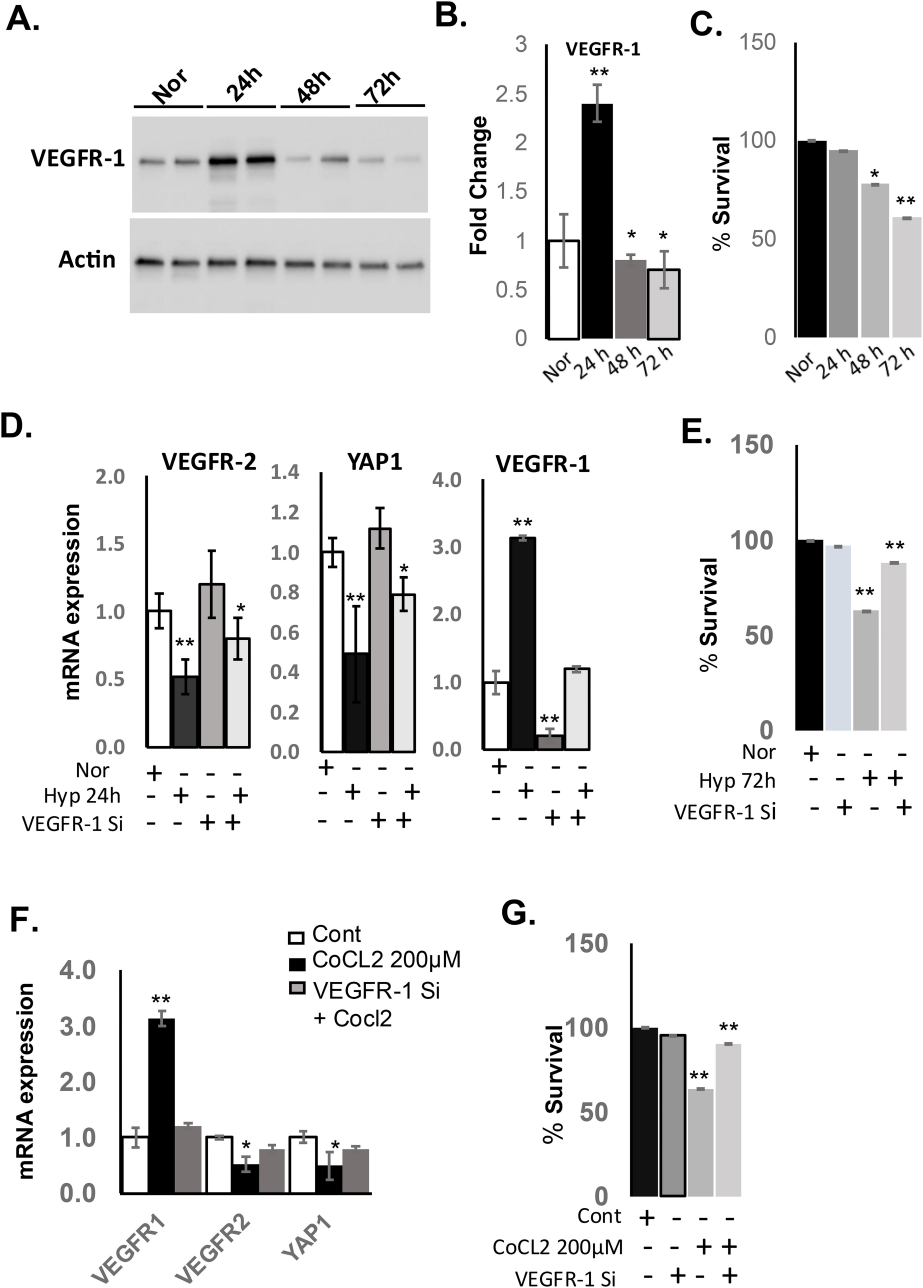
Hypoxia induces VEGFR1-mediated neuronal death in SH-SY5Y cells. **(A)** Representative western blot image showing expression of VEGFR-1 when SH-SY5Y cells were exposed to hypoxia (hyp, 1%O_2_) for 24, 48, and 72 h. **(B)** Quantification showing the fold change of the VEGFR-1 protein expression from **(A)**. **(C)** MTT assay showing increased neuronal death in SH-SY5Y cells after hypoxia (hyp, 1%O_2_) treatment for 24, 48, and 72 h compared to normoxia (Nor). **(D)** mRNA expression of VEGFR-1, VEGFR-2, and YAP1 in SH-SY5Y cells after 24 h hypoxia treatment (hyp, 1%O_2_) and VEGFR-1 siRNA treatment. **(E)** MTT assay showing increased cell survival in SH-SY5Y cells after VEGFR-1 siRNA treatment 24 h prior to 72 h hypoxia treatment (hyp, 1%O_2_). **(F)** mRNA expression of VEGFR-1, VEGFR-2 and YAP1 in SH-SY5Y cells after hypoxia treatment (24 h, CoCL2, 200 μM) and VEGFR-1 knockdown prior to 24 h hypoxia treatment (CoCL2, 200 μM). **(G)** MTT assay showing increased cell death in SH-SY5Y cells exposed to hypoxia (CoCL2,200 μM) for 72 h and increased cell survival after siRNA-mediated VEGFR-1 knockdown prior to CoCL2 treatment. Mean ± SD. *p < 0.05, **p < 0.01 (Student’s *t*-test), *n* = *3* independent experiments.

To further explore a potential detrimental effect of increased VEGFR-1 expression in neurons, we first exposed SH-SY5Y human neuroblastoma cells to hypoxia conditions (1%O_2_) for 24, 48, and 72 h. Our results show that hypoxia exposure significantly upregulated VEGFR-1 expression in the SH-SY5Y cells after 24 h hypoxia (Figures 6A,B), however, these increased VEGFR-1 levels significantly declined after 48 and 72 h of hypoxia treatment (Figures 6A,B), likely due to neuronal toxicities, which we confirmed using MTT assays, which showed increased toxicities/cell death particularly after 72 h of hypoxia exposure (Figure 6C). We also confirmed that 24 h hypoxia treatment significantly increased VEGFR-1 mRNA levels (Figure 6D), whereas mRNA levels of cell survival genes e.g., VEGFR-2 and Hippo pathway YAP1 levels, were significantly reduced in the SH-SY5Y cells (Figure 6D). We next tested whether siRNA-mediated VEGFR-1 knockdown in the SH-SY5Y cells could prevent cellular toxicity induced by hypoxia treatment. To do this, the SH-SY5Y cells were treated with VEGFR-1 siRNA for 24 h prior to either 24 or 72 h hypoxia exposure. We show that siRNA mediated knock down of VEGFR-1 rescued both YAP1 and VEGFR-2 mRNA levels after 24 h hypoxia treatment (Figure 6D) and importantly, prevented neuronal toxicities/cell death following 72 h hypoxia exposure (as measured by MTT assays, Figure 6E). We then further validated these results using Cobalt Chloride (CoCL_2_) treatment (200 μM for 24 h) as an additional method of inducing hypoxia conditions in the SH-SY5Y cells. We confirmed that CoCL_2_ treatment for 24 h increased VEGFR-1 expression and reduced both VEGFR-2 and Hippo pathway YAP1 levels in the SH-SY5Y cells (Figure 6F) and siRNA-mediated knock down of VEGFR-1 prior to treatment with CoCL_2_ restored both YAP1 and VEGFR-2 mRNA levels (Figure 6F) and prevented neuronal toxicities/cell death (as measured by MTT assays, Figure 6G), implicating aberrant VEGFR-1 expression and signaling in hypoxia-induced neuronal toxicities/cell death.

**FIGURE 7:**
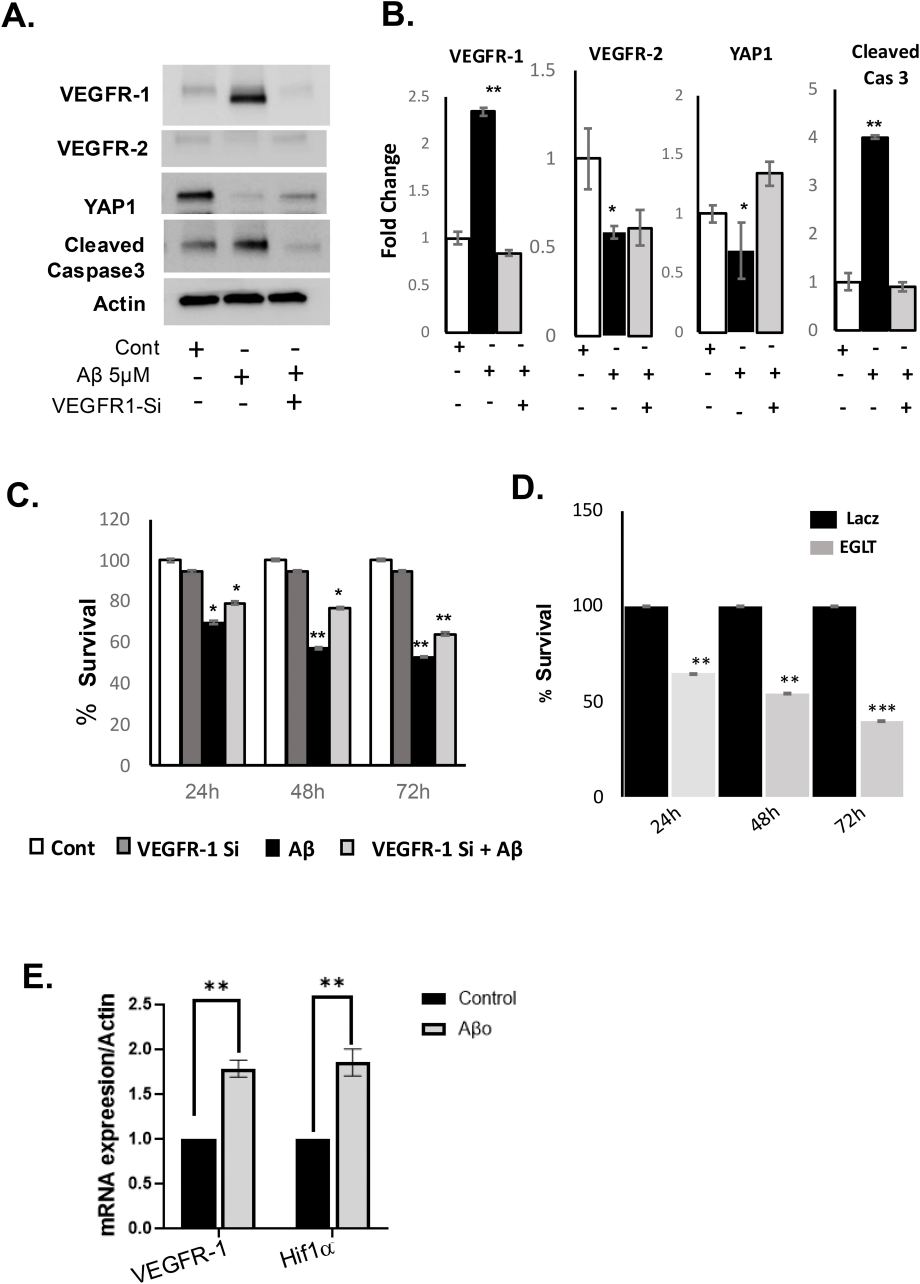
VEGFR-1 knockdown prevents Ab 1-42 oligomer -induced neuronal death in SH-SY5Y cells. **(A)** Representative western blot image showing increased protein levels of VEGFR-1 and cleaved caspase3 and decreased VEGFR-2 and YAP1 levels in response to Ab1-42 oligomers (Ab, 5 μb, 5esentative west h in SH-SY5Y cells and siRNA mediated VEGFR-1 knockdown partially restored VEGFR-2 and YAP-1 levels. **(B)** Quantification of western blot bands from **(A)** showing fold change of the protein levels. **(C)** MTT assay showing decreased cell survival in the SH-SY5Y neurons in response to Ab1-42 oligomers (Ab, 5 μb, 5ssay showing decreased ce, 48, and 72 h and increased survival after siRNA mediated VEGFR-1 knockdown. **(D)** MTT assay showing increased neuronal toxicities/ cell death in the SH-SY5Y neurons in response to transfection with VEGFR-1 “EGLT” construct for 24, 48, and 72 h. **(E)** mRNA expression of VEGFR-1 and HIF1-a in SH-SY5Y cells after 24 h Ab1-42 oligomers (Abo, 5 μβ?, 5 ?λιγ?μερMean ± SD. *p<0.05, ** *p* < 0.01, ***p < 0.0001 (Student’s *t*-test). *n* = 3 independent experiments.

We next examined the effects of Aβ1-42 oligomer (5 μM) treatment on VEGFR-1 expression in the SH-SY5Y cells. Treatment of SH-SY5Y cells with the Aβ1-42 oligomers for 24 h readily increased VEGFR-1 and cleaved caspase 3 protein levels (as measured by western blot, Figures 7A,B), leading to enhanced neuronal toxicities/cell death (as measured by MTT assays, Figure 7C). Similar to hypoxia treatment, both VEGFR-2 and YAP1 levels were also significantly reduced in the SH-SY5Y cells after 24 h Aβ1-42 oligomers treatment (Figures 7A,B). We also demonstrate that siRNA-mediated knockout of VEGFR-1 in the SH-SY5Y neurons restored both YAP1 and VEGFR-2 protein levels, reduced cleaved caspase 3 protein levels (Figures 7A,B) and significantly prevented the Aβ1-42 oligomer induced neuronal toxicities/cell death (as measured by MTT assays, Figure 7C), again implicating VEGFR-1 expression and signaling in the induction of neuronal toxicities/cell death pathways in response to Aβ1-42 oligomers. To further evaluate cell death phenotypes, we treated SH-SY5Y cells with 5 μM Aβ1-42 oligomers for 72 h and quantified Apopxin staining using confocal microscopy. Compared to control cells, Aβ1-42 oligomer treated cells exhibited a marked (∼10 fold) increase in green fluorescence, indicating significant apoptosis induction (Supplementary Figures 2A,B). Importantly, siRNA-mediated VEGFR-1 knockdown significantly reduced the Aβ1-42 oligomer-induced apoptotic signaling, as evidenced by decreased Apopxin fluorescence intensity (Supplementary Figures 2A,B). Similarly, using Flow cytometry analysis of Annexin V and propidium iodide (PI) staining, we show that SH-SY5Y cells treated with 5 μM Aβ1-42 oligomers for 72 h, showed marked increase in apoptotic cells (∼35–40%) (Supplementary Figures 2C,D), whereas siRNA-mediated VEGFR-1 knockdown significantly reduced apoptotic cell populations in Aβ1-42 oligomer treated SH-SY5Y cells (Supplementary Figures 2C,D).

To determine whether over-expression of the VEGFR-1 in the SH-SY5Y cells can directly induce neuronal toxicities, we used a previously characterized construct in which the extracellular N-terminal domain of VEGFR-1 is deleted and replaced with the extracellular domain of epidermal growth factor receptor (EGFR) fused to the transmembrane and intracellular domains of VEGFR-1 (referred to as “EGLT”), where signaling is mediated exclusively through the VEGFR-1 intracellular domains (Zeng et al., 2001; Singh Angom et al., 2019). We show that transfection with the VEGFR-1 “EGLT” construct for 24, 28, and 72 h in SH-SY5Y cells led to significantly increased neuronal toxicities/cell death in all time points analyzed compared to LacZ transfection control (as measured by MTT assays, Figure 7D), indicating that over-expression of VEGFR-1 intracellular domains was sufficient to induce neuronal toxicities in the SH-SY5Y cells. We next wondered whether oxidative stress generated in response Aβ1-42 oligomers may influence HIF-1α expression in the SH-SY5Y cells, potentially contributing to increased VEGFR-1 expression in response to Aβ1-42 oligomer treatment. Previous studies have documented that hypoxia conditions can increase hypoxia inducible factors including HIF-1α, which can increase transcription of VEGFs including VEGFR-1 to regulate angiogenesis in ECs (Krock et al., 2011; Wicks and Semenza, 2022; Bae et al., 2024). And previous studies have also shown that Aβ oligomers can readily increase reactive oxygen species (ROS) (Kayed and Lasagna-Reeves, 2013; Cheignon et al., 2018; Butterfield and Halliwell, 2019) in neurons and increased production of ROS can influence HIF-1α stability and functions (Cash et al., 2007). In our study, we show that increased mRNA expression of HIF-1α was observed in the SH-SY5Y neuronal cells treated with Aβ1-42 oligomers for 24 h (Figure 7E), suggesting that the increased HIF-1α expression could potentially regulate VEGFR-1 expression in this paradigm.

**FIGURE 8:**
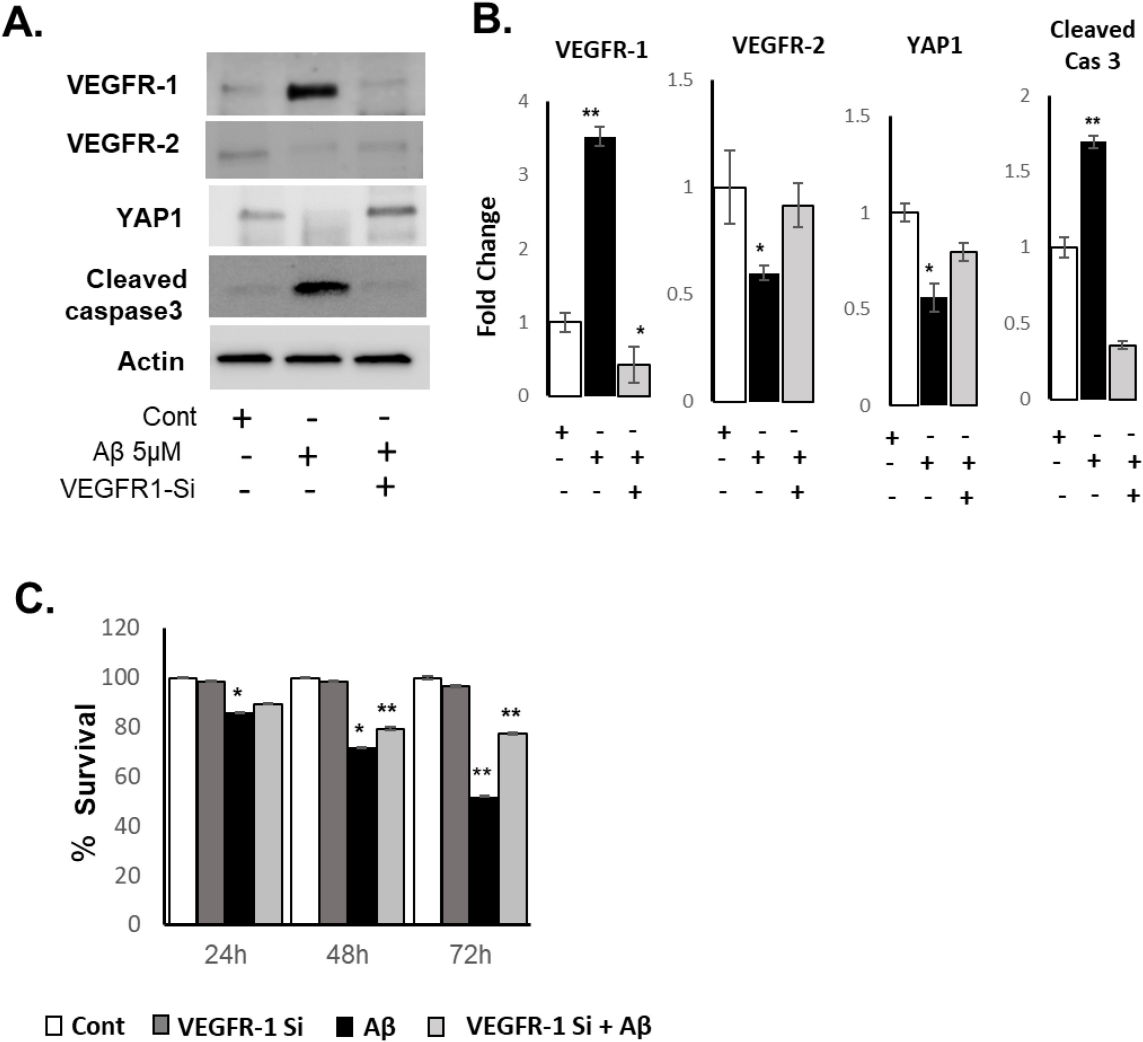
VEGFR-1 knockdown prevents Aβ 1-42 oligomer induced neuronal death in mouse primary neurons. **(A)** Representative western blot image showing increased protein levels of VEGFR-1 and cleaved caspase3 and decreased VEGFR-2 and YAP1 levels in response to ?β1-42 oligomers treatment (Aβ, 5 μM) for 24 h in the mouse primary neurons and siRNA-mediated VEGFR-1 knockdown partially restored VEGFR-2 and YAP-1 levels. **(B)** Quantification of western blot bands from **(A)** showing fold change of the protein levels. **(C)** MTT assay showed decreased cell survival in the mouse primary neurons in response to ?β1-42 oligomers treatment (Aβ, 5 μM) for 24, 48, and 72 h) and increased survival after siRNA-mediated VEGFR-1 knockdown. Mean ± SD. *p < 0.05, **p < 0.01, (Student’s *t*-test). *n* = *2* independent experiments.

We further validated the above findings using *mouse primary neurons* (Mouse CD1 Brain Cortex Neurons, Lonza). Treatment with 5 μM Aβ1-42 oligomers for 24 h readily increased VEGFR-1 protein levels, reduced VEGFR-2 and YAP1 protein levels and increased cleaved caspase 3 levels in the mouse primary neurons, (as measured by western blot, Figures 8A,B), precipitating in increased neuronal toxicities/cell death (as measured by MTT assays, Figure 8C). We also confirmed that siRNA-mediated knockdown of VEGFR-1 rescued both YAP1 and VEGFR-2 protein levels, prevented cleaved caspase activation (Figures 8A,B), and reduced neuronal toxicities/cell death after Aβ1-42 oligomers treatment in the mouse primary neurons (Figure 8C). We also observed increased VEGFR-1 as well as cleaved caspase 3 levels in SH-SY5Y cells treated with Aβ1-42 fibrils for 24 h (5 μM, as measured by western blot, Supplementary Figures 3A,B), whereas treatment of SH-SY5Y cells for 24 h with Aβ1-42 monomers (5 μM), did not increase VEGFR-1 or cleaved caspase 3 protein levels (Supplementary Figures 3A,B), suggesting that this effect is specific to Aβ1-42 oligomeric species and Aβ1-42 fibrils. Treatment of the SH-SY5Y cells with Aβ1-42 fibrils (5 μM and 10 μM) for 72 h also induced robust neuronal toxicities/cell death (as measured by MTT assays, Supplementary Figure 3C). Similar to above results, we again demonstrate that siRNA-mediated knockout of VEGFR-1 in the SH-SY5Y cells significantly reduced neuronal toxicities/cell death after 72 h of Aβ1-42 fibril treatment (as measured by MTT assays, Supplementary Figure 3C), also implicating aberrant VEGFR-1 expression and signaling events in activation of neuronal cell death pathways in response to Aβ1-42 fibrils.

### 3.3 Aβ1-42 oligomer exposure induces oxidative stress and neuro-inflammatory gene expression in neurons.

**FIGURE 9:**
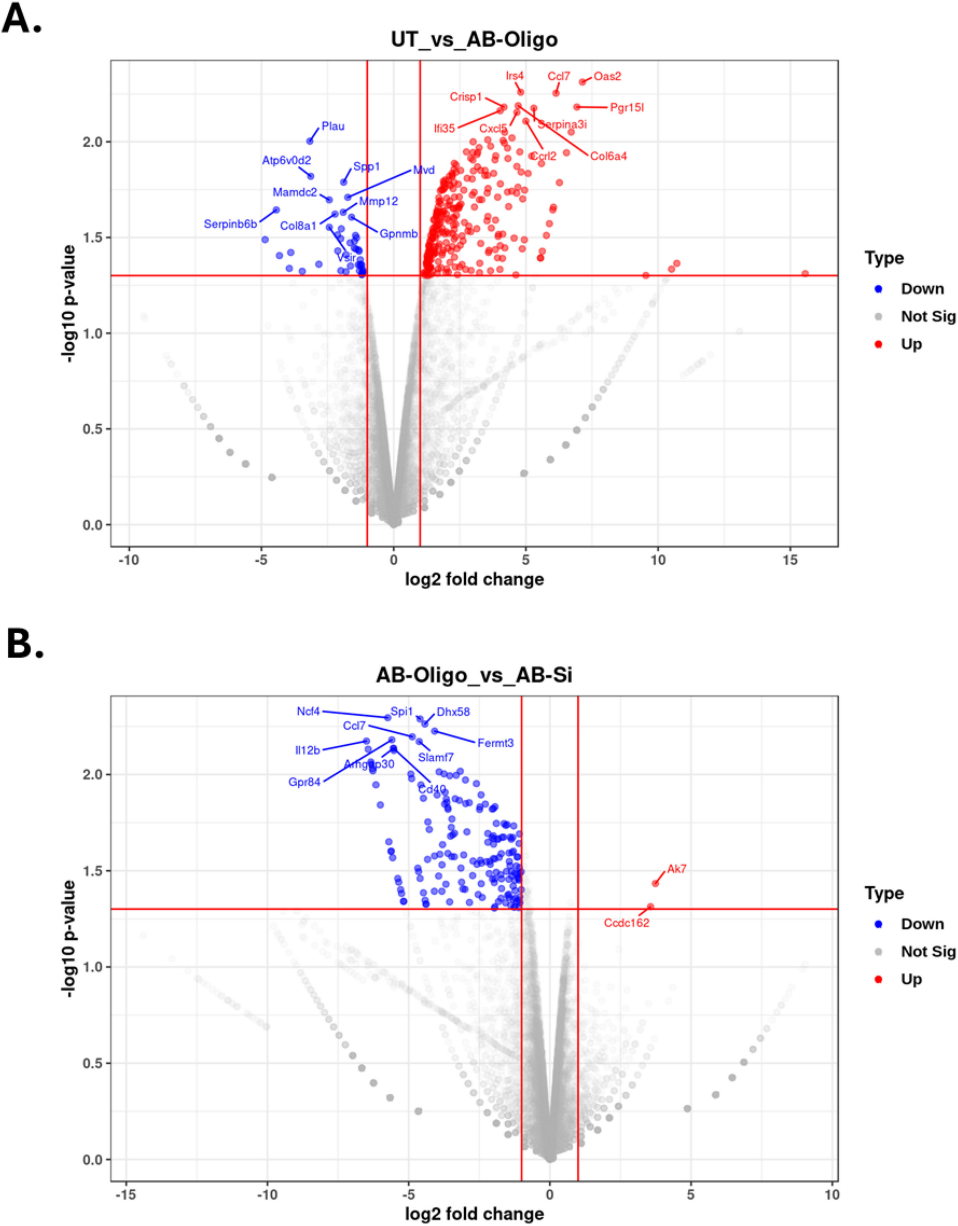
Differential gene expression profiles in ?β1-42 oligomers treated mouse primary neurons. **(A)** Representative volcano plot showing both upregulated and downregulated genes in the neurons following ?β1-42 oligomer treatment for 24 h (AB-Oligo) compared to untreated controls (UT). **(B)** Representative volcano plot showing both upregulated and downregulated genes in the Aβ1-42 oligomer treated neurons after siRNA-mediated VEGFR-1 knockdown (AB-Si). Red- Upregulated Genes. Blue- Downregulated genes.

To examine the transcriptomic effects of Aβ1-42 oligomer induced neuronal cell death, we performed bulk RNA sequencing in mouse primary neurons treated with Aβ1-42 oligomers for 24 h. Analysis of the top 50 differentially regulated genes identified a significant number of genes that were either upregulated or downregulated in response to Aβ1-42 oligomers treatment (*p*-value < 0.05; |log2 fold change| > 1; Figures 9A,B and Table 1). Analysis of the *upregulated genes* in the Aβ1-42 oligomer-treated neurons showed significant enrichment in pathways involving neuro-inflammatory responses (Figure 9A). For example, genes such as *Nlrc5, Trim21, Ifi27, Irf9, Ifitm3, Parp12*, and *Zc3hav1* pointed toward an interferon-stimulated gene (ISG) response. Gene ontology (GO) and pathway enrichment analysis confirmed the pronounced activation of neuro-immune associated pathways, and the anti-viral transcriptional signature in the neurons in response to Aβ1-42 oligomers treatment (see Supplementary Figure 4A). Similarly, several genes involved in apoptosis and oxidative stress were significantly upregulated including *Txnrd1, Prdx1, Gadd45b and Casp3*, indicating that Aβ induces a neuroimmune activation signature and pro-apoptotic signaling. Neuronal activity and synaptic function also appeared impacted, as evidenced by upregulation of immediate-early genes such as *Egr1, Egr2, Egr3, Junb, Npas4*, and *Id1*, as well as neurotransmission-related genes like *Gad1, Gabrq, Slc17a7*, and *Adcyap1*, suggesting activity-dependent transcriptional remodeling in response to Aβ1-42 oligomer induced stress. In addition, the mouse primary neurons treated with Aβ1-42 oligomer exhibited significant *downregulation* of genes critical for maintaining lipid metabolism, neuronal integrity, and immune homeostasis (Figure 9B). A prominent signature of suppressed genes included those involved in cholesterol biosynthesis and lipid regulation, such as *Mvd, Fdps, Scd1, Acat2, Dhcr7*, *Hmgcs1, Mvk*, and *Stard4*. The repression of these genes indicates a disruption in neuronal lipid and membrane homeostasis, a hallmark of Aβ oligomer-induced toxicities. Gene ontology (GO) and pathway enrichment analysis confirmed the pronounced downregulation of the neuronal lipid and membrane homeostasis genes in the neurons in response to Aβ1-42 oligomers treatment (Supplementary Figure 4B). Similarly, genes critical for extracellular matrix (ECM) stability and neuronal structural support, such as *Mmp12, Col8a1, Fmod, Prelp*, and *Fbln5*, were also repressed, potentially compromising the structural integrity of the neurons. Other notable downregulated genes included certain AD associated genes, including e.g., *Tes, Plau*, and *Lrp2.* Collectively, these transcriptional changes reveal that Aβ1-42 oligomer exposure not only activates oxidative stress and neuro-inflammatory responses but also suppresses key metabolic and structural programs in the neurons.

**TABLE 1:** Modulation of *upregulated* genes following Aβ1-42 oligomer treatment by siRNA-mediated VEGFR-1 knockdown.

Upregulated genes	Aβ(1-42)		Aβ(1-42) + VEGFR-1 Si
	Fold change	*P*-value	Fold change
*Cd14*	2.997154639	0.011378186	−0.030
*Cybb*	1.515474892	0.038269181	−0.050
*Il1rap*	1.395450379	0.045211798	
*Nlrc5*	2.070456615	0.04797841	−0.352
*Trim21*	2.296919791	0.04006894	−0.337
*Irf9*	1.578945407	0.035223581	−0.114
*Ifitm3*	2.750103986	0.011290390	−3.0147
*Parp12*	2.24902979	0.017597679	−2.520
*Zc3hav1*	2.262801305	0.027209517	−0.646
*Ccl9*	3.004251942	0.010003894	−7.341
*Lgals9*	2.993099182	0.012448254	−5.751
*Gadd45b*	2.032433888	0.021533132	−0.373
*Hmox1*	1.880644729	0.016092702	−2.479
*Dusp1*	1.397126194	0.045266726	−0.980
*Chchd10*	1.902407047	0.02309212	0.339
*Npas4*	2.073243675	0.015865994	
*Id1*	2.106559036	0.021373972	−3.846
*Gad1*	1.528439398	0.025738308	
***Flt1***	**1.6752278011**	**0.012759498**	−**3.992**

Bold values indicate regulation of “genes of interest”.

### 3.4 VEGFR-1 silencing in neurons suppresses pro-inflammatory and stress-related gene expression in response to Aβ1-42 oligomers

In parallel to above experiments, to assess the potential role of VEGFR-1 in modulating these responses, we also analyzed gene expression patterns in the neurons treated with Aβ1-42 oligomers after siRNA-mediated VEGFR-1 knockdown. VEGFR-1 silencing resulted in broad downregulation of genes associated with inflammation, apoptotic stress, and redox regulation, indicating a potential anti-inflammatory and neuroprotective effect (Figure 9B). Several key mediators of immune signaling were suppressed, including *Tlr1, Tlr3, Syk, Csf1r*, *Ripk2*, *Nfkb2*, *ifitm3, Irf1, Lcp2, C4b*, and *Itgb2*, suggesting attenuation of innate immune receptor signaling, inflammasome activity and broad suppression of interferon-stimulated pathways. Similarly, expression of key pro-apoptotic regulators including *Casp1, Prkcd, Myc*, and *Parp10* were also reduced in addition to reduced expression of reactive oxygen species (ROS)-responsive genes *Sqor, Blvrb*, and *Gusb*, also suggesting that VEGFR-1 knockdown reduced activation of apoptotic cell death pathways in response to Aβ1-42 oligomers. Gene ontology (GO) and pathway enrichment analysis confirmed downregulation of the neuro-inflammatory responses in the VEGFR-1 siRNA treated neurons as well as downregulation of tyrosine kinase signaling pathways in response to Aβ1-42 oligomers treatment (Supplementary Figure 4A). Furthermore, using this analysis, we also show that gene expression in specific interferon-induced inflammatory and oxidative stress related genes that were highly upregulated in response to Aβ1-42 oligomers treatment, (e.g., *Parp12, Ccl9, Ifitm3, and Hmox1)*, were significantly downregulated in the VEGFR-1 siRNA treated neurons (Table 1). Similarly, in some genes that were significantly downregulated following Aβ1-42 oligomers treatment (e.g., *Lrp2*), their expression was rescued following VEGFR-1 silencing (Table 2), although this was not the case for other genes (e.g., *Plau*), whose expression levels were not altered following VEGFR-1 silencing in the neurons. Using this gene expression analysis, we also confirmed significantly increased expression of VEGFR-1/*Flt1* following Aβ1-42 oligomers treatment (Table 1) and reduced expression VEGFR-2/*Kdr* and *Yap1* levels in the Aβ1-42 oligomer treated mouse primary neurons (Table 2). These reduced levels of VEGFR-2/*Kdr* and *Yap1* were partially rescued in the VEGFR-1 siRNA treated neurons (Table 2). Taken together, these data highlight the potential neuro-protective role of VEGFR-1 in modulation of neuro-inflammatory and oxidative stress-responsive apoptotic cell death programs in the neurons in response to Aβ-1-42 oligomer exposure.

**TABLE 2:** Modulation of *downregulated* genes following Aβ1-42 oligomer treatment by siRNA-mediated VEGFR-1 knockdown.

*Downregulated genes*	Aβ(1-42)		Aβ(1-42) + VEGFR-1 Si
	Fold change	*P-*value	Fold change
*Mvd*	−1.736666078	0.019512441	0.3004
*Fdps*	−1.648587138	0.033720096	
*Scd1*	−1.192776699	0.046248138	0.0002
*Dhcr7*	−1.263116608	0.0412949	0.091
*Hmgcs1*	−1.156444939	0.047331465	0.257
*Stard4*	−1.186984896	0.047928488	0.457
Mfge8	−1.444188253	0.030834879	
Itgax	−1.800082437	0.038894908	
Cd24a	−1.207626659	0.044028849	
*Fmod*	−1.981582705	0.028488533	0.39
*Prelp*	−1.805233764	0.047735076	0.159
*Plau*	−3.170252222	0.009929867	
*Lrp2*	−2.114347273	0.037149646	1.226
***Kdr***	−**0.8004549915**	**0.29857273**	**0.681**
***Yap1***	−**0.16554537333**	**0.24402609**	**0.621**

Bold values indicate regulation of “genes of interest”.

## 4 Discussion

Recent data using single cell/nuclei transcriptome analysis have demonstrated increased VEGFR-1 levels in various cells types, including ECs, neurons and microglial cells in AD brains, and increased expression levels of VEGFR-1 correlated with worse disease outcomes (Mahoney et al., 2019; Seto et al., 2023; Mahzarnia et al., 2024), suggesting a potential detrimental role of increased/aberrant VEGFR-1 expression on AD associated pathologies. However, these transcriptome data do not explain whether the increased/aberrant VEGFR-1 expression is the cause or consequence of AD pathology. In this study, using paraffin-embedded fixed AD brain sections, we demonstrated VEGFR-1 immunoreactivity in the neuropil and in neuronal cell bodies in the hippocampus and strong immunoreactivity in ependymal cells lining the choroid plexus but weak EC staining in the AD samples. Using anti-VEGFR-1 antibodies with C-terminal epitopes, we show robust VEGFR-1 immunoreactivity in the vicinity of ubiquitin positive dystrophic neurites, specifically near “cored” Thio-S positive amyloid plaques both in AD brains and in aged Tg2576 mice. Increased VEGFR-1 immunoreactivity in neuritic process near amyloid plaques was also recently reported in aged CRND8 mouse brains (Levites et al., 2024). Although recent RNA seq data have shown increased VEGFR-1 expression in multiple cell types in AD brain, including microglia cells and ECs, our immuno-histological analysis using 5 μM paraffin sections showed weak EC immunoreactivity in the hippocampus and did not show any immunostaining in microglial cells, however, a previous report (using 30 μM free floating sections) reported increased VEGFR-1 immuno-reactivity in AD brain microglial cells (Ryu et al., 2009). Using multiple different VEGFR-1 antibodies, our data showed noticeably different staining patterns in the AD brain, raising concerns regarding VEGFR-1 specificity and/or epitope preferences, as such additional comparative analysis and methods of tissue preparation e.g., differential effects of paraffin embedding vs. frozen brain tissue will be necessary to further validate these immuno-histological findings. In addition, using both SH-SY5Y cells and mouse primary neurons, we demonstrate that treatment with either hypoxia conditions (1%O_2_ or 200 μM CoCl_2_) or 5 μM Aβ1-42 oligomers, resulted in increased VEGFR-1 expression and cleaved caspase 3 activation, precipitating in neuronal toxicities and cell death/apoptosis. Similarly, increased VEGFR-1 and cleaved caspase-3 protein levels and neuronal toxicities were also readily observed in 5 and 10 μM Aβ1-42 fibril-treated SH-SY5Y cells. Importantly, siRNA-mediated knockout of VEGFR-1 in the neurons prevented the hypoxia, Aβ1-42 oligomer and Aβ1-42 fibril-induced neuronal toxicities/apoptosis, indicating a potential detrimental role of increased VEGFR-1 expression and signaling events in neurons in response to AD pathologies.

Using gene expression analysis, we demonstrated significant transcriptional changes characterized by activation of oxidative stress/cell death pathways and enrichment of immune-related pathways including anti-viral interferon-stimulated gene (ISG) expression in response to Aβ1-42 oligomers in the mouse primary neurons. Indeed, upregulation in these pathways including upregulation of interferon responses have been recently demonstrated in AD brains in response to both amyloid and tau pathology (Taylor et al., 2014; Hur et al., 2020; Roy et al., 2020; Sanford and McEwan, 2022; Sanford et al., 2024). Importantly, we show that the expression of these interferon-induced genes that were upregulated in response to Aβ1-42 oligomers, were significantly blunted following siRNA-mediated VEGFR-1 knockdown, highlighting the potential neuro-protective role of VEGFR-1 in modulating neuro-inflammatory and oxidative stress responses in neurons in response to Aβ1-42 oligomer exposure. However, we recognize an important limitation of this data, which is that we did not include biological replicates for analysis at the time of sequencing, as such these data should be considered exploratory in nature and warrant further validation experiments. In addition, since these data show significant increases in gene expression of immune-related genes, we do not exclude the possibility of contamination of non-neuronal cells, in particular glial cells/astrocytes that are often isolated together with the primary neurons, which may skew the analysis.

Recent transcriptome data have demonstrated decreased VEGFR-2 expression in AD brains (Seto et al., 2023; Mahzarnia et al., 2024) and decreased VEGFR-2 levels have also been reported in brains of Tg2575 mice (Cho et al., 2017), suggesting that AD pathologies could trigger VEGFR-2 depletion. In addition, increases in soluble VEGFR-1 (sVEGFR1) levels, could also potentially block VEGFR-2 signaling (Risau, 1997; Grunewald et al., 2021), leading to detrimental effects in the neurons. In our study, we show that VEGFR-2 levels in both SH-SY5Y cells and mouse primary neurons were reduced following either hypoxia or Aβ1-42 oligomer treatment and these reduced levels were partially restored by siRNA-mediated VEGFR-1 knockdown. Similarly, previous studies have demonstrated YAP-1 dysregulation in brains of AD mouse models (Xu et al., 2018; Tanaka et al., 2020). In our study, we show that in both SH-SY5Y cells and mouse primary neurons exposed to either Aβ1-42 oligomers or hypoxia conditions, YAP-1 levels were significantly reduced and these levels were normalized after VEGFR-1 knockdown, suggesting potential beneficial effects of siRNA-mediated VEGFR-1 knockdown on both VEGFR-2 and YAP-1 expression levels in the neurons.

Mechanistically, we show that over-expression of VEGFR-1 by itself, using the VEGFR-1 “EGLT” construct, readily induced neuronal toxicities/cell death, indicating that increased/aberrant expression and signaling event mediated by the VEGFR-1 intracellular domains may have a significant role in directly inducing neuronal toxicities/cell death. In this regard, previous studies have demonstrated γ-secretase mediated processing of intracellular VEGFR-1 C-terminal domains following ligand stimulation both in ECs and leukemia cells (Rahimi et al., 2009; Cai et al., 2011; Silva et al., 2021). And multiple lines of evidence suggest that the intracellular cleavage products generated following γ-secretase cleavage contain transcription-activation domains and protein-interaction domains, indicating the main biological function of these γ-secretase substrates is in transcriptional regulation (Kopan and Ilagan, 2004; Hur, 2022). Thus, one possibility is that γ-secretase generated VEGFR-1 intracellular domains in the neurons may could transcriptionally regulate cell survival gene expression and/or activation of cell death pathways in the neurons, but this needs further examination. Our hypothesis is that both hypoxia conditions in the brain (in areas of ischemia//BBB damage) or local high concentrations of soluble Aβ oligomers or fibrils (in the vicinity/penumbra of amyloid plaques (Koffie et al., 2009; Condello et al., 2015; Yang et al., 2017)) induce oxidative stress/neuro-inflammation and activation of HIFs, leading to increased expression of both VEGFR-1 and its ligands, resulting in increased expression and aberrant signaling of VEGFR-1, leading to activation of cell death pathways in neurons and suggest that modulation of VEGFR-1 expression and signaling could prevent neuronal cell death. Indeed, beneficial effects of modulating VEGFR-1 expression/signaling in endothelial cells (ECs) have recently been demonstrated in various disease models. For example, using either genetic knockout or antibody mediated inhibition of endothelial VEGFR-1 showed a marked increase of vascular density, improved metabolism, and improved muscle function in a mouse model of Duchenne muscular dystrophy (Bosco et al., 2021) and in mice fed with high-fat diet (Seki et al., 2018). Similarly, our group previously demonstrated that VEGFR-1 knockdown in the ECs prevented both hypoxia and Aβ1-42 oligomer induced EC senescence (Kulkarni et al., 2019; Singh Angom et al., 2019; Angom et al., 2022). Lastly, anti-VEGFR-1 antibody treatment led to neuroprotective responses in Aβ1-42 injected in rat hippocampus by modulation of microglial chemotactic responses (Ryu et al., 2009). These findings, together with our current work, demonstrate that modulation of VEGFR-1 expression can have potential beneficial responses in AD brain and establish a link with aberrant VEGFR-1 expression and signaling in AD pathogenesis. In future studies, we aim to further examine the potential transcriptional regulatory role of VEGFR-1 by further investigating signaling events of VEGFR1 and its ligands as well as identifying their potential binding partners and/or interactions with other transcription factors, which may lead to future development of VEGFR-1 specific small molecule inhibitors, which are currently not available in the clinic.

In conclusion, these data establish a prominent role of aberrant VEGFR-1 expression and signaling on activation of neuronal toxicities/cell death pathways in response to hypoxia, Aβ1-42 oligomers and fibrils and provide additional insight regarding the recent RNA seq data, which show increased VEGFR-1 expression in AD brain cells associated with worse disease outcomes (Mahoney et al., 2019; Lau et al., 2020; Seto et al., 2023; Mahzarnia et al., 2024). Importantly, our data show that modulating VEGFR-1 expression and signaling in neurons significantly prevented both hypoxia and Aβ1-42-induced cellular toxicities/cell death and suggest that modulating VEGFR-1 expression and signaling may have beneficial effects on AD associated pathologies.

## Data Availability

The gene expression data generated in this study have been deposited in the NCBI Sequence Read Archive (SRA) under accession number PRJNA1415957.

## Footnotes

1
http://bioconductor.org/packages/release/bioc/html/DESeq2.html
