# Correction: Vascular endothelial growth factor receptor-1 (VEGFR-1) knock-down is protective against hypoxia, Aβ1-42 oligomer and Aβ1-42 fibril-induced neuronal cell death: implications in AD pathogenesis

**DOI:** 10.3389/fnins.2026.1901421

**Published:** 2026-07-07

**Authors:** Ramcharan Singh Angom, Zonghua Li, Hari Krishnareddy Rachamala, Monica Castanedes-Casey, Tanmay Kulkarni, Trisha Chakravarty, Shamit Dutta, Enfeng Wang, Dennis Dickson, Debabrata Mukhopadhyay, Pritam Das

**Affiliations:** 1 Department of Biochemistry and Molecular Biology, Mayo Clinic Florida, Jacksonville, FL, United States; 2 Department of Neuroscience, Mayo Clinic Florida, Jacksonville, FL, United States

**Keywords:** Alzheimer's disease, Aβ oligomers/fibrils, hypoxia, neuronal cell death, VEGFR-1

The order of authors in the author list of the published paper was erroneously given as:

Debabrata Mukhopadhyay, Pritam Das, Ramcharan Singh Angom, Shamit Dutta, Zonghua Li, Monica Castanedes-Casey, Tanmay Kulkarni, Trisha Chakravarty, Enfeng Wang, Dennis Dickson and Hari Krishnareddy Rachamala.

The correct author list reads:

Ramcharan Singh Angom, Zonghua Li, Hari Krishnareddy Rachamala, Monica Castanedes-Casey, Tanmay Kulkarni, Trisha Chakravarty, Shamit Dutta, Enfeng Wang, Dennis Dickson, Debabrata Mukhopadhyay, Pritam Das.

The original version of this article has been updated.

