# Supplementary material for: Vascular endothelial growth factor receptor-1 (VEGFR-1) knock-down is protective against hypoxia, Aβ1-42 oligomer and Aβ1-42 fibril -induced neuronal cell death: implications in AD pathogenesis
Source: Front Neurosci. 2026 May 29;20:1799391. doi: 10.3389/fnins.2026.1799391 (PMC13260607; doi:10.3389/fnins.2026.1799391)
Supplement: Supplementary file 1 [file Supplementary_file_1.pdf]

Supplementary Figure 1.

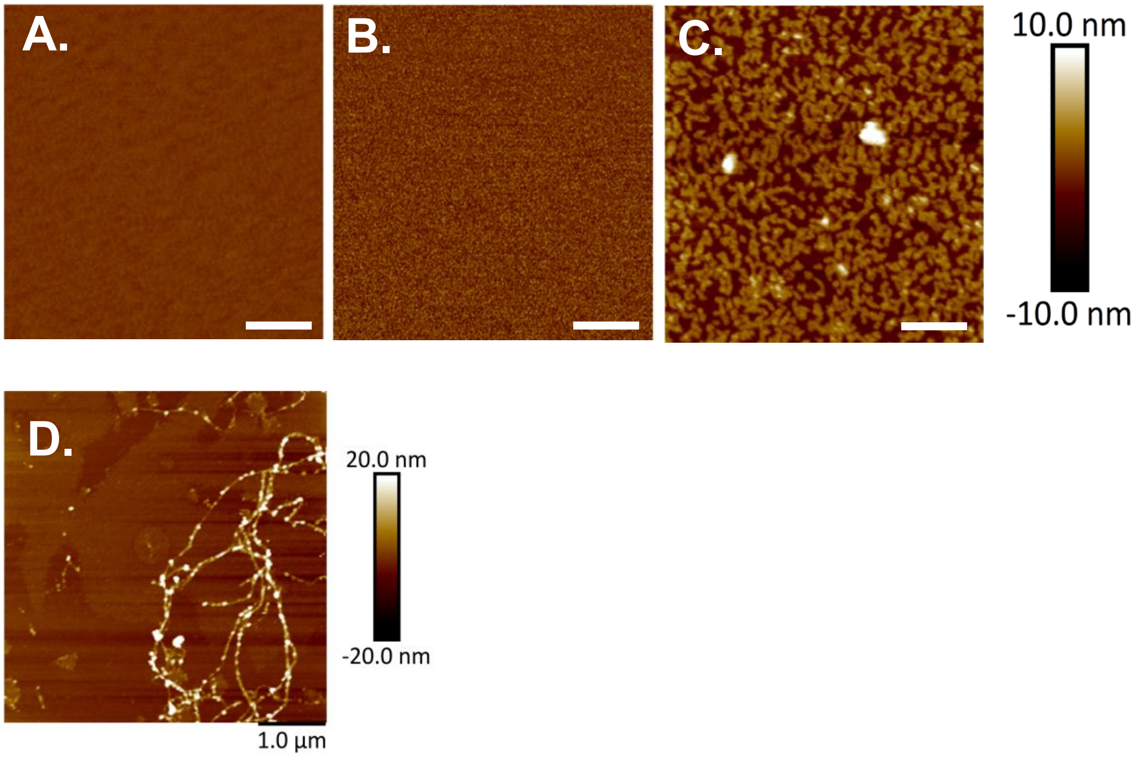

Supplementary Figure 1. Representative Image of (**A**). Plain mica; (**B**). APTES modified mica; (**C**) Aβ 1-42 oligomers (2.5 μM). Scale bar = 200 nm. (**D**) Aβ 1-42 Fibrils (2.5 μM).

Supplementary Figure 2.

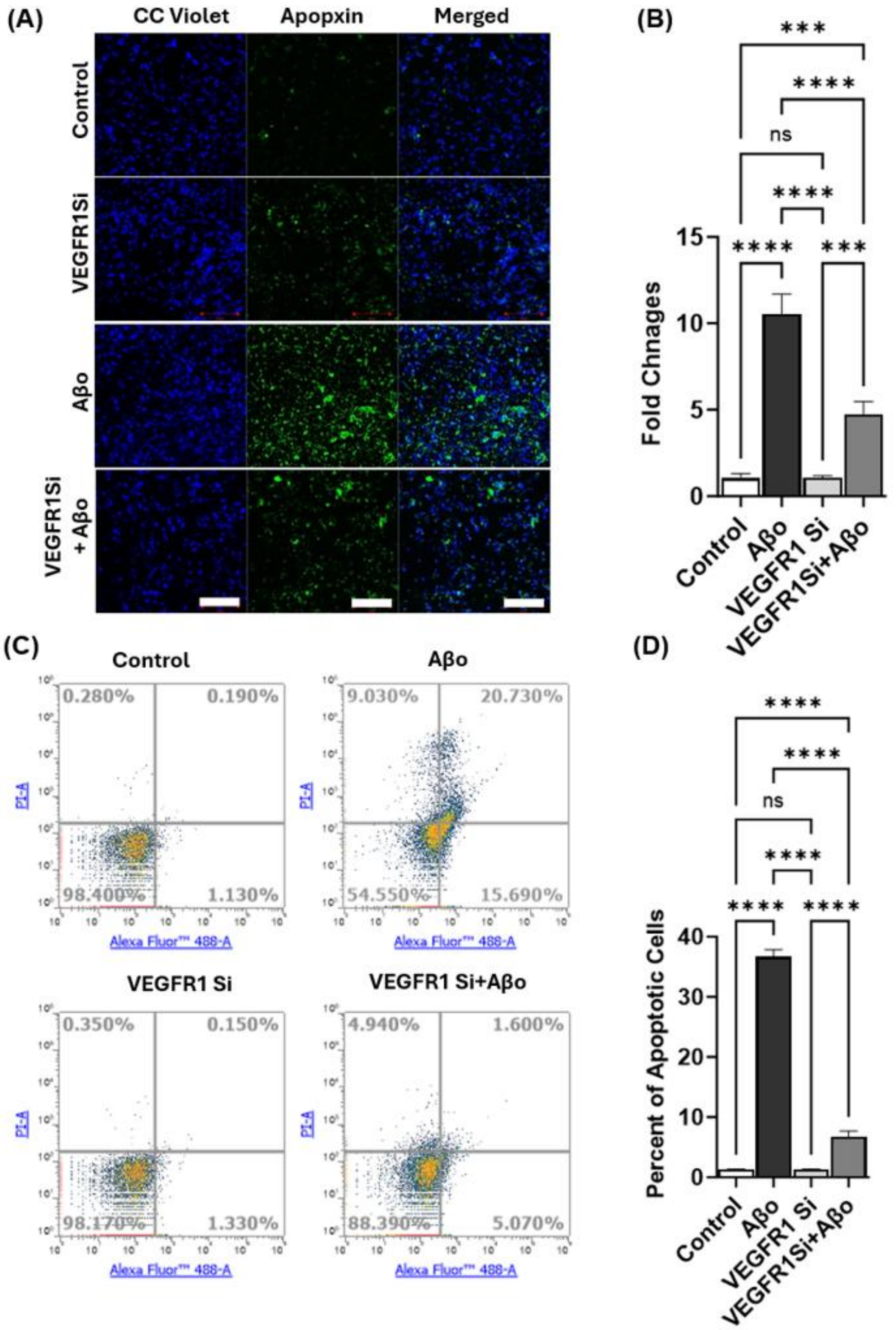

Supplementary Figure 2. VEGFR-1 silencing attenuates Aβ1-42 oligomer-induced apoptosis in SH-SY5Y cells. (**A**) Representative confocal images of SH-SY5Y cells stained with Apopxin Green (apoptotic cells) and Calcein Violet (blue) in Control, Aβ1-42 oligomer (Aβo, 5μM), VEGFR-1 siRNA (VEGFR1 Si), and VEGFR1 Si + Aβo treated SH-SY5Y cells. (**B)** Quantification of Apopxin fluorescence intensity (represented as fold change relative to Control). (**C**) Representative flow cytometry dot plots of Annexin V (Alexa Fluor™ 488) and propidium iodide (PI) staining showing viable, early apoptotic, and late apoptotic cell populations. (**D**) Percentage of total apoptotic cells (early + late apoptosis). Data are presented as mean ± SEM (*n* = 3). Statistical significance was determined by one-way ANOVA. ***$p < 0.001$, ****$p < 0.0001$, ns = not significant. *Scale bar = 200 μm.*

Supplementary Figure 3.

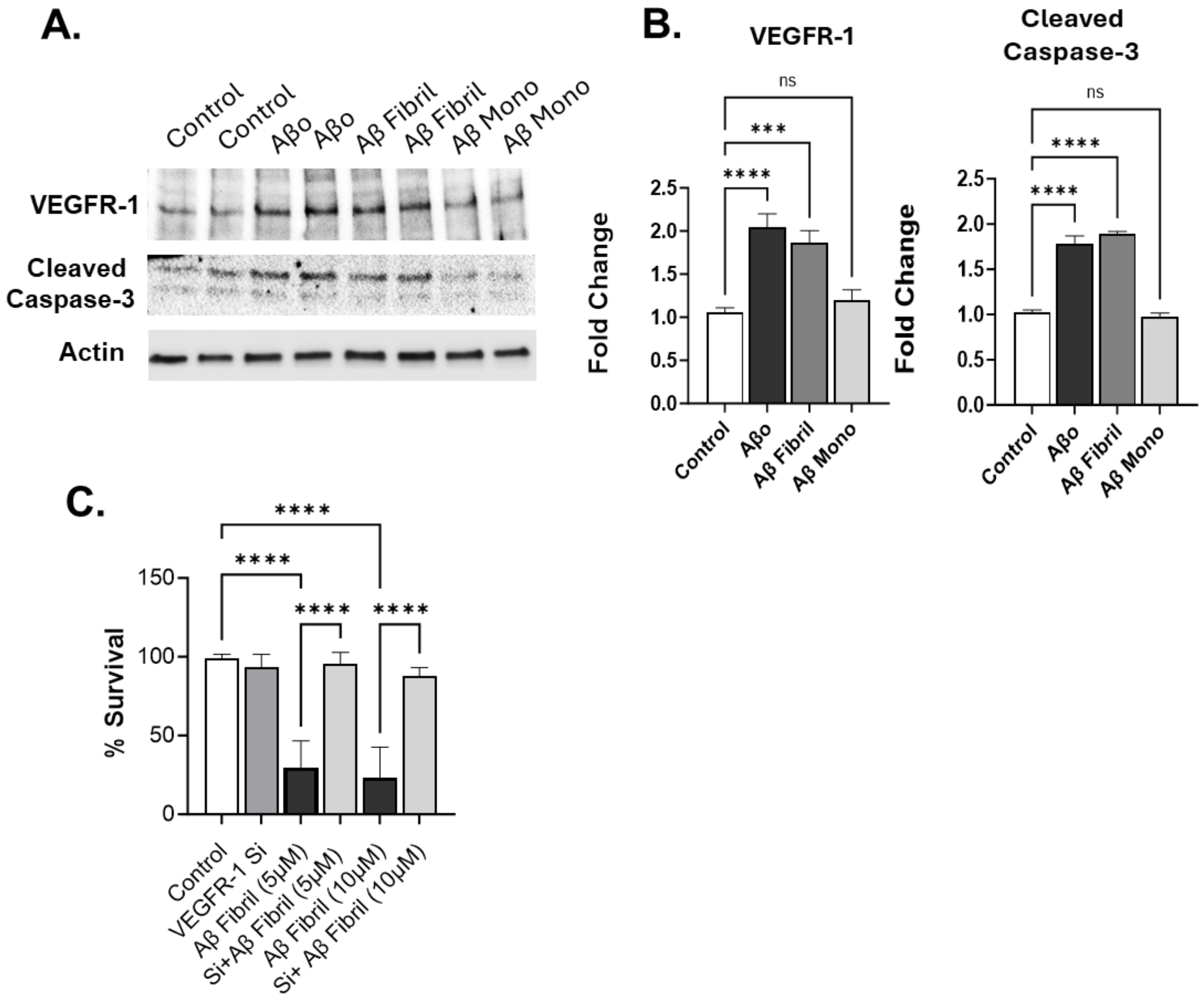

Supplementary Figure 3. VEGFR-1 knockdown prevents Aβ 1-42 fibril induced neuronal death in SH-SY5Y cells. **A**. Representative western blot image showing increased protein levels of VEGFR-1 and cleaved caspase-3 in response to Aβ1-42 oligomers (Aβo, 5μM) and Aβ1-42 fibrils (Aβ fibril, 5μM) but not Aβ1-42 monomers (Aβ Mono, 5μM) treatment for 24hrs in SH-SY5Y cells. **B**. Quantification of western blot bands from (A) showing fold change of the protein levels. **C.** MTT assay showing increased cell death in the SH-SY5Y neurons in response to Aβ1-42 fibrils treatment for 72hrs (Aβ fibril, 5μM and 10μM) and robust cell death prevention after siRNA-mediated VEGFR-1 knockdown (VEGFR-1 Si). Data represented as Mean ± SD. ***, $p<0.001$, **** $p < 0.0001$, (One-way ANOVA). *n = 2 independent experiments.*

Supplementary Figure 4.

(A)

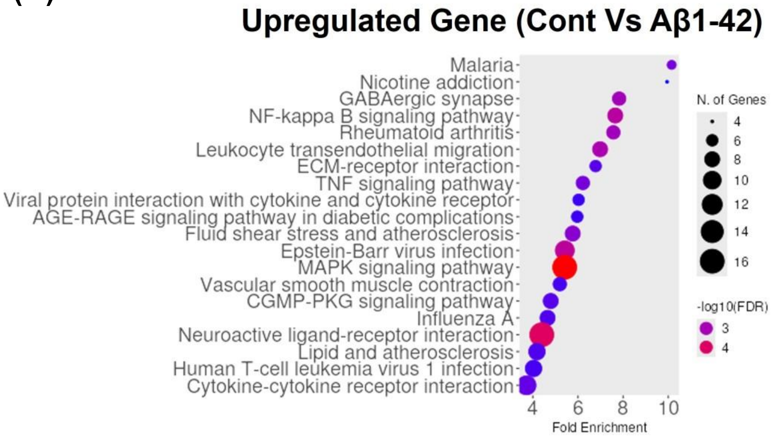

(B)

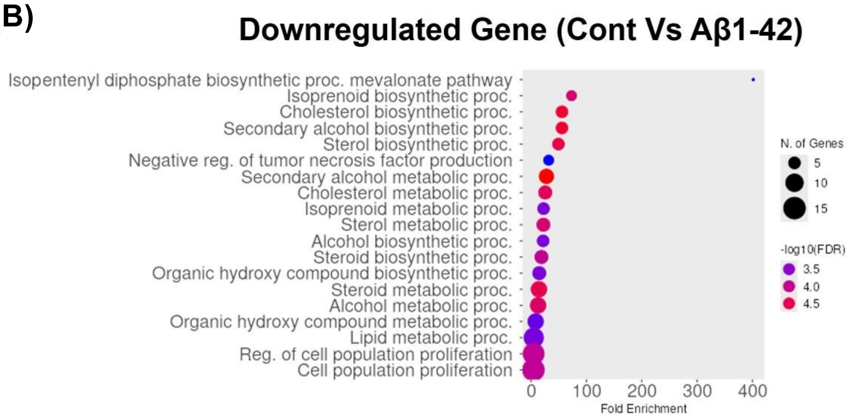

(C)

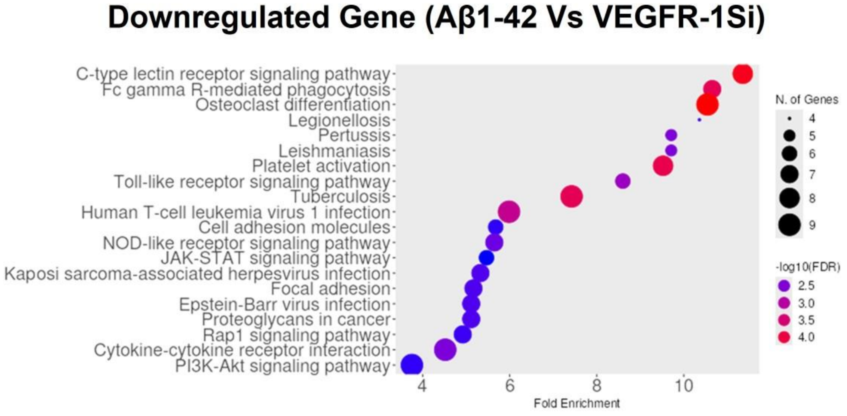

Supplementary Figure 4. KEGG-based gene set enrichment analysis in Aβ1-42 oligomer - treated mouse primary neurons. (A) Dot plots showing the top 20 enriched KEGG terms associated with significantly upregulated genes in the Aβ1-42 oligomer treated neurons compared to untreated controls. **(B)** Dot plots showing the top 20 enriched KEGG terms associated with significantly down-regulated genes in the Aβ1-42 oligomer treated cells compared to untreated controls. **(C)** Dot plots showing the top 20 enriched KEGG terms associated with significantly down-regulated genes in the Aβ1-42 oligomer treated neurons after SiRNA-mediated VEGFR-1 knockdown.
